# Unraveling ocean $\hbox {pCO}_{2}$ dynamics in Northwest Greenland Fjords

**DOI:** 10.1038/s41598-025-12720-1

**Published:** 2025-08-11

**Authors:** Camille Hayatte Akhoudas, Adam Ulfsbo, Brett F. Thornton, John W. Pohlman, Lee-Gray Boze, Martin Jakobsson, Christian Stranne

**Affiliations:** 1 Department of Geological Sciences, Stockholm University, Stockholm, Sweden; 2 Bolin Centre for Climate Research, Stockholm University, Stockholm, Sweden; 3 Department of Marine Sciences, University of Gothenburg, Gothenburg, Sweden; 4 U.S. Geological Survey, Woods Hole Coastal and Marine Science Center, Woods Hole, MA USA

**Keywords:** Greenland fjords, Surface ocean pCO_2_, Freshwater input influence, Biological CO_2_ uptake, Biogeochemistry, Climate sciences, Ocean sciences

## Abstract

We investigated the relative contributions of various factors that influence seasonal changes in sea surface partial pressure of $\hbox {CO}_{2}$ ($\hbox {pCO}_{2}$, calculated from the measured pH and total alkalinity) in four regions of northwestern Greenland: Nares Strait, Lincoln Sea, Sherard Osborn and Petermann fjords. Using the temperature minimum layer as a proxy for winter conditions, we examined $\hbox {pCO}_{2}$ dynamics from the onset of sea-ice melt to summer. Our findings revealed significant spatial variability in $\hbox {pCO}_{2}$, driven by differences in temperature, freshwater inputs, and biological activity. In particular, in Sherard Osborn Fjord substantial freshwater inputs and strong stratification were found to enhance $\hbox {pCO}_{2}$ accumulation, while in Petermann Fjord biological $\hbox {CO}_{2}$ uptake was the main driver. This study, conducted in summer 2019, underscores the critical role of northwest Greenland’s coastal waters as a summer $\hbox {CO}_{2}$ sink. It highlights the complex interplay of physical and biogeochemical processes in modulating $\hbox {pCO}_{2}$, suggesting significant regional differences in $\hbox {CO}_{2}$ dynamics between two neighboring fjords.

**Supplementary Information:**

The online version contains supplementary material available at 10.1038/s41598-025-12720-1.

## Introduction

The Arctic Ocean is warming up to four times faster than the global average^1^, triggering a cascade of environmental changes with profound implications, such as a sharp decrease in sea-ice cover^2–4^ and increased glacial meltwater inputs into the surface ocean^5–7^. These transformations are expected to have complex repercussions on the exchange of $\hbox {CO}_{2}$ between the atmosphere and the ocean^8–12^, a pivotal process in global climate regulation. Although it covers only 4 % of the Earth’s ocean area, the Arctic Ocean is a disproportionate player in the global carbon cycle, accounting for 5-14 % of global $\hbox {CO}_{2}$ fluxes^13–15^. However, substantial uncertainties persist due to the challenges of accurately estimating $\hbox {CO}_{2}$ fluxes in the temporally and spatially undersampled Arctic Ocean^16^.

**Fig. 1:**
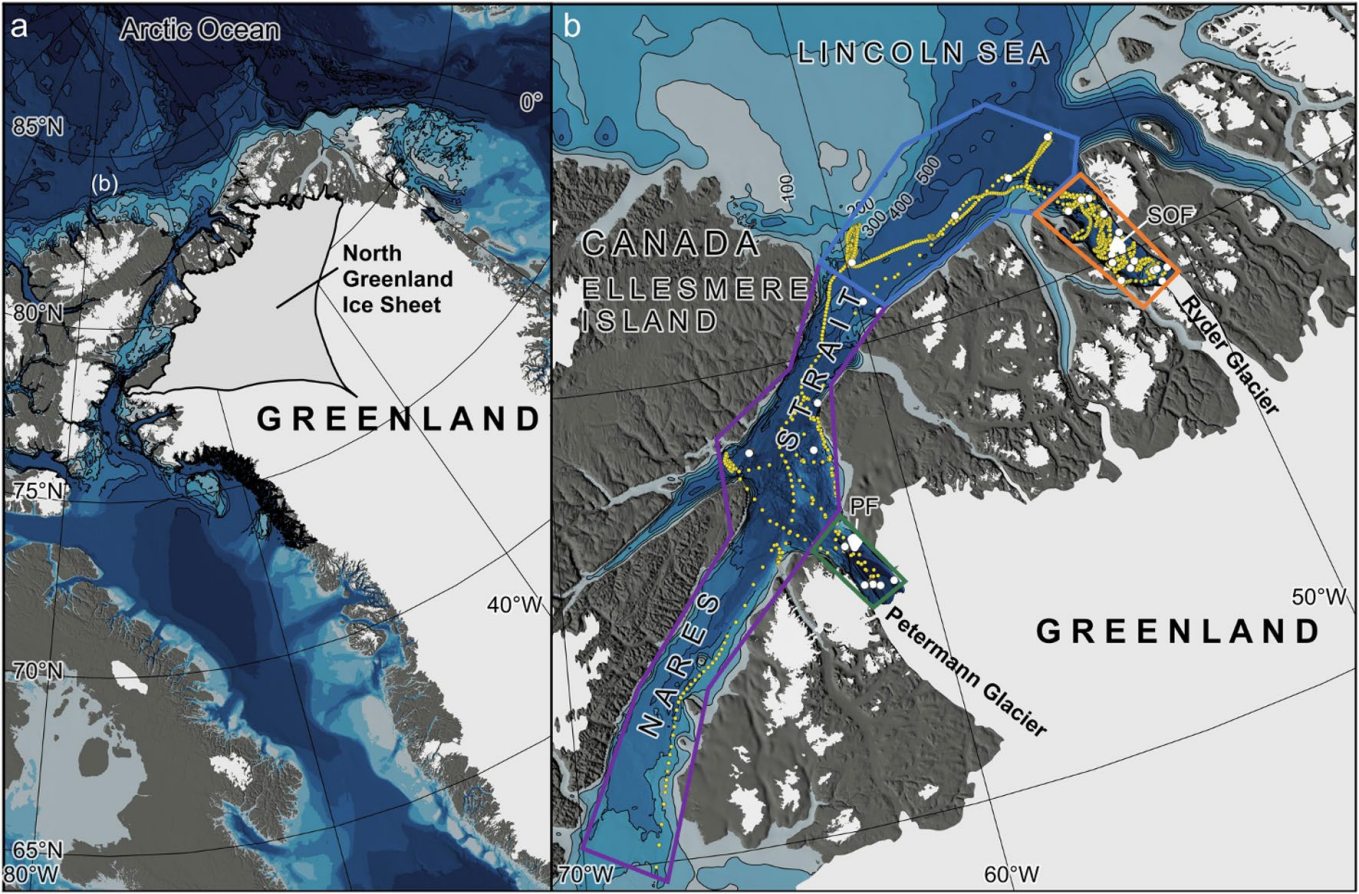
Maps of the study area. The drainage sector of the North Greenland Ice Sheet as defined by ref.^121^ is shown in a, while the detailed map in b shows the track of underway measurements in focus for this study. The bathymetry is from the International Bathymetric Chart of the Arctic Ocean (IBCAO) Version 4.0^122^. PF corresponds to Petermann Fjord (81$^{\circ }$N, 61$^{\circ }$W) and SOF corresponds to Sherard Osborn Fjord (82$^{\circ }$N, 51$^{\circ }$W). The white dots show the locations of the CTD stations. The map was made using QGIS (Version 3.34.2 Prizren).

The Northwest of Greenland is home to several large outlet glaciers that drain the North of Greenland ice sheet^17,18^, along with a continental shelf area known for its heavy sea-ice conditions^19^. In this study, we present hydrographic and biogeochemical data acquired during the *Ryder 2019* expedition with Swedish icebreaker *Oden* from the Nares Strait, the Lincoln Sea, Petermann Fjord, and the never-before surveyed Sherard Osborn Fjord (Fig. 1a). These datasets provide the opportunity to investigate surface ocean $\hbox {pCO}_{2}$ in the Sherard Osborn and Petermann Fjords, where the waters are influenced by marine-terminating glaciers that ultimately discharge into the Lincoln Sea and the Nares Strait, respectively. Although these neighboring fjords share similarities, their distinct hydrographies^20^ and local sea-ice conditions result in varying impacts on their marine physical and biogeochemical characteristics^21^.

**Fig. 2:**
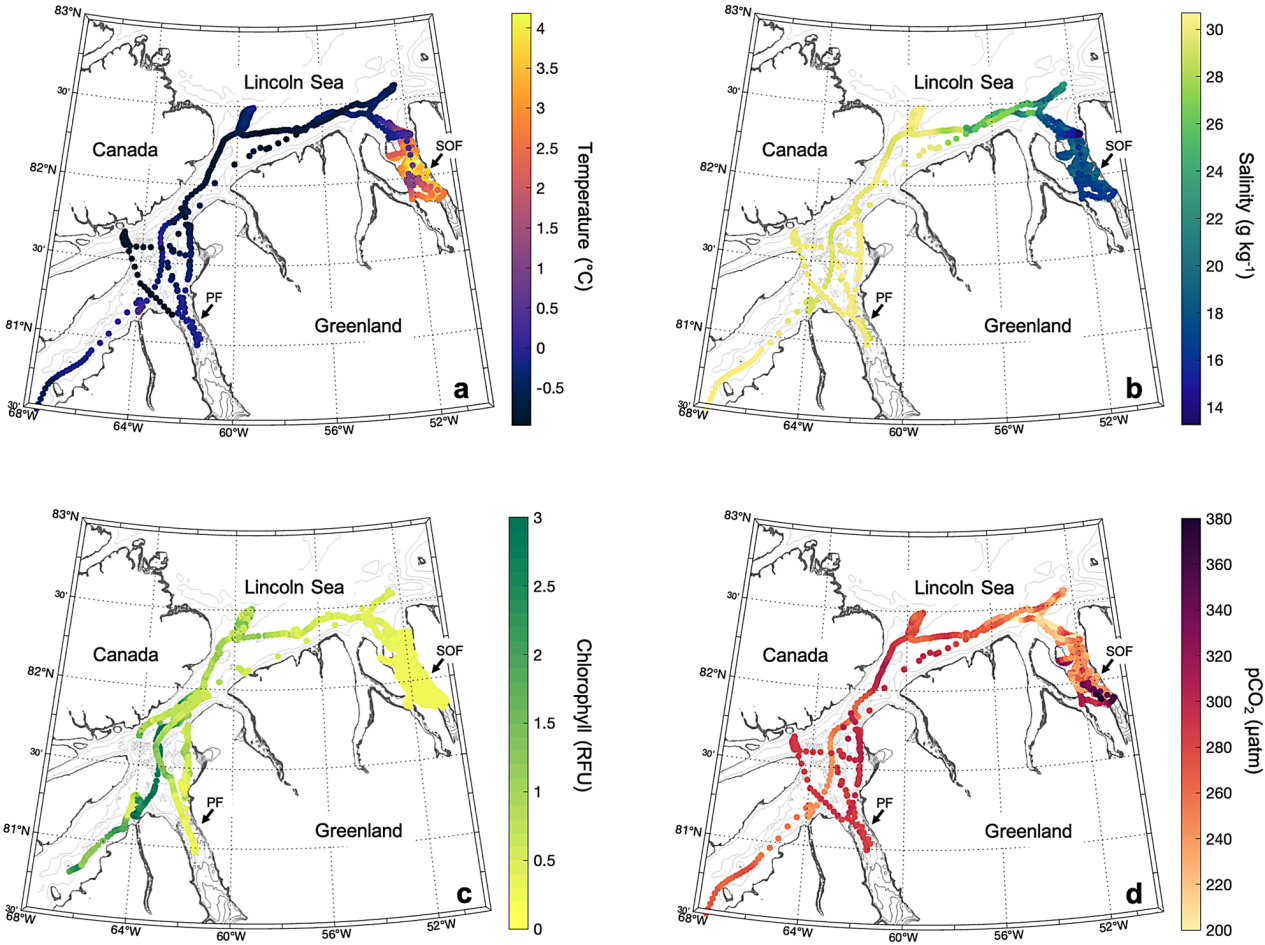
Spatial distribution of underway measurements of surface seawater salinity (**a**), temperature (**b**), chlorophyll *a* (**c**) and $\hbox {pCO}_{2}$ (**d**) of the study area. PF corresponds to Petermann Fjord and SOF corresponds to Sherard Osborn Fjord.

Limited research has been conducted on the $\hbox {CO}_{2}$ system in northwestern Greenland^22–26^, and to our knowledge, this is the first work to present high-frequency $\hbox {pCO}_{2}$ data from the Lincoln Sea, Sherard Osborn Fjord, and Petermann Fjord. Previous studies in glacier-influenced Greenland fjords suggest that freshwater inputs significantly lowers surface water $\hbox {pCO}_{2}$, leading to high $\hbox {CO}_{2}$ uptake^27–31^. For example, in coastal waters of East Greenland, where primary production is low^32,33^, relatively stable $\hbox {pCO}_{2}$ levels seem to be primarily influenced by freshwater inputs^30,34^. In contrast, the highly productive coastal waters of western Greenland (5–42 mol C $\hbox {m}^{-2}$ $\hbox {yr}^{-1}$) suggest a greater role for biological processes in carbonate dynamics and $\hbox {CO}_{2}$ uptake, though freshwater inputs remain a contributing factor^28,32,34,35^. However, the relative contributions of biological activity and freshwater inputs remain uncertain, as do the mechanisms through which freshwater inputs influence $\hbox {pCO}_{2}$ in these shelf waters restricted by the characteristics of the fjords. Given Greenland’s highly heterogeneous coastline, understanding the drivers of $\hbox {pCO}_{2}$ in surface waters is essential for assessing regional $\hbox {CO}_{2}$ uptake. Furthermore, ice-free Arctic Ocean basins have been postulated to increase atmospheric $\hbox {CO}_{2}$ uptake^8,10,13^. Here, we apply a simple model that uses days since ice retreat (DSR) as a temporal reference, relative to the onset of the open water period. This approach has been used to examine how sea ice-dependent processes–such as air-sea $\hbox {CO}_{2}$ fluxes, surface warming, and biological production–affect $\hbox {pCO}_{2}$ after sea-ice retreats, e.g. refs^8,10,12,36,37^. These studies show that post-DSR, increased $\hbox {pCO}_{2}$ from surface warming and air-sea $\hbox {CO}_{2}$ exchange outweighs reductions due to biological production and freshwater inputs, leading to elevated sea surface $\hbox {pCO}_{2}$. This suggests that prolonged ice-free periods will further raise $\hbox {pCO}_{2}$, likely diminishing the Arctic Ocean’s role as a net $\hbox {CO}_{2}$ sink^10,36,37^. The resulting decrease in surface water alkalinity and buffering capacity could lead to sharp pH declines^8,12^, with significant biological consequences at regional scales^8^.

In this context, our study focuses on the sensitivity of surface water $\hbox {pCO}_{2}$ to environmental constraints from the onset of sea-ice melting, during which winter surface water starts to interact with the atmosphere, until the end of summer. Our measurements reveal that the surface waters of the region were undersaturated in $\hbox {CO}_{2}$ relative to the atmosphere. Although surface waters in Petermann Fjord remained relatively homogeneous in terms of salinity, temperature, $\hbox {pCO}_{2}$, and air-sea $\hbox {CO}_{2}$ flux, Sherard Osborn Fjord exhibited a wider range of these parameters, with notably concomitant low $\hbox {pCO}_{2}$ (as low as 180 $\mu$atm) and higher $\hbox {pCO}_{2}$ values (exceeding 300 $\mu$atm) compared to other areas. We investigate the underlying mechanisms driving these variations, and in particular the influences of biotic and abiotic factors on the evolution of surface $\hbox {pCO}_{2}$. Ultimately, our study underscores the critical role of surface ocean warming and freshwater inputs in shaping the future $\hbox {CO}_{2}$ sink of Greenland fjords in a warming climate.

## Data and Methods

### Underway measurements

Underway measurements of seawater pH^38^ and total alkalinity (TA)^39^ were taken every 10 min using spectrophotometric techniques (Contros HydroFIA pH and TA, -4-H Jena engineering GmbH, Kiel, Germany) from the ship’s underway system (bow intake at 8 m depth). Seawater pH was measured at S = 35 and $\hbox {T} = 25\,^{\circ }\hbox {C}$ on the total scale. TA was measured at S = 35 and $\hbox {T} = 25\,^{\circ }\hbox {C}$, and calibrated by routine calibrations of certified reference material (CRM Batch *#181*) obtained from A. G. Dickson of Scripps Institution of Oceanography (La Jolla, CA, USA). Both the seawater pH and TA data were post-processed and recalculated using the ship’s underway salinity (SBE45) and temperature (SBE45) data. Chlorophyll (fluorescence in RFU) was continuously measured by a YSI Inc. EXO2 multiparameter sonde and recorded at 30 second intervals. Atmospheric $\hbox {CO}_{2}$ was continuously measured with a cavity ring-down laser spectrometer (Model 0010, FGGA 24EP, Los Gatos Research (LGR), Mountain View, California, USA). Raw measurements were collected at 1 Hz. $\hbox {CO}_{2}$ precision is 0.25 ppm; accuracy is 0.25 ppm. Although the laser spectrometer exhibits low drift, instrumental drift was nonetheless determined by measurement of target gases of known concentration every 8 hours; these measurements were used to correct for the small drift in the data. The target gases were themselves cross-calibrated pre and post-cruise with calibrated standard gases obtained from the NOAA CMDL, based on whole air collected at Niwot Ridge, Colorado. We computed surface seawater partial pressure of CO_2_ (hereafter $\hbox {pCO}_{2}^{sw}$) using TA and pH data (supplementary Figure 1) with MATLAB CO2SYS version 1.2^40^. This involved applying stoichiometric dissociation constants for carbonic acid ($\hbox {K}_{1}$ $^{*}$ and $\hbox {K}_{2}$ $^{*}$) and bisulfate ($\hbox {K}_{HSO4}$ $^{*}$) as provided by ref.^41^, refit by ref.^42^, and ref.^43^, respectively. The uncertainty of these calculations was estimated using the subroutines of the CO2SYS program, which propagate assumed errors of 5 $\mu$mol $\hbox {kg}^{-1}$ for TA and 0.01 for pH. These error estimates are appropriate for uncertainty propagation, as outlined by ref.^44^, especially given that duplicate measurements were not systematically conducted during the expedition–except for HydroFIA pH, the uncertainty was determined to be 0.005 ± 0.003 pH units, based on the absolute differences of seawater sample duplicates (n = 15). Uncertainties for other parameters involved in the calculations–such as temperature, salinity, and the dissociation constants–were assigned default values from ref.^44^.

### CTD and sampling

Observations of temperature and salinity were made during the Ryder 2019 Expedition using a Seabird 911 CTD (conductivity, temperature, depth). The CTD was equipped with a 22 Niskin bottle (12 liters) rosette and the following sensors: Dual SeaBird temperature (SBE 3) and conductivity (SBE 04C), dissolved oxygen (SBE 43), turbidity, and fluorescence (WET Labs ECO-AFL/FL) and a Benthos Altimeter PSA-916D. In situ conductivity and temperature have been converted to conservative temperature and absolute salinity using the TEOS-10 equation of state.

**Fig. 3:**
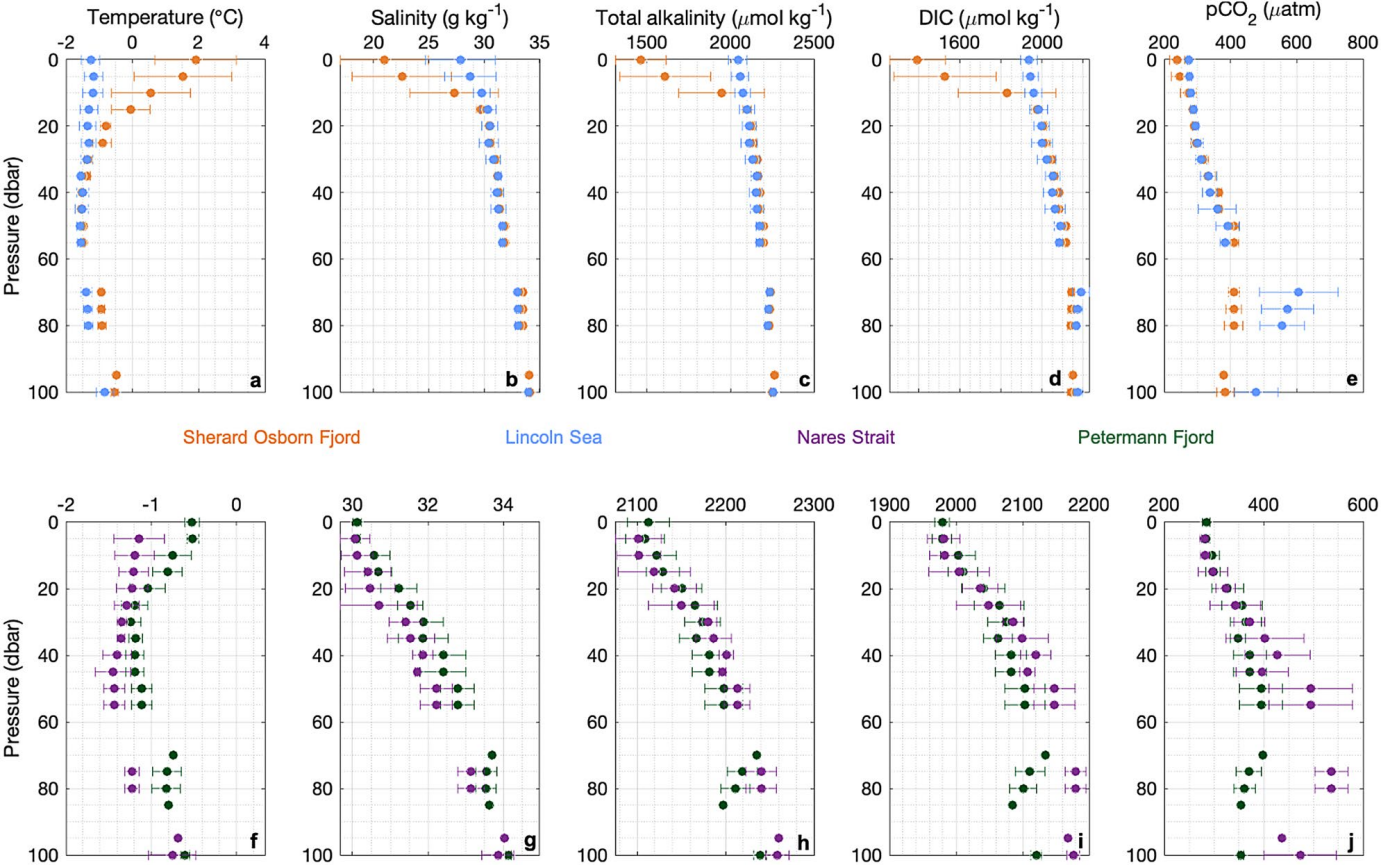
Vertical profiles of seawater temperature (**a**,**f**), salinity (**b**,**g**), TA (**c**,**h**), DIC (**d**,**i**) and $\hbox {pCO}_{2}$ (e,j) in Sherard Osborn Fjord (orange), Petermann Fjord (green), the Nares Strait (purple) and the Lincoln Sea (blue).

**Fig. 4:**
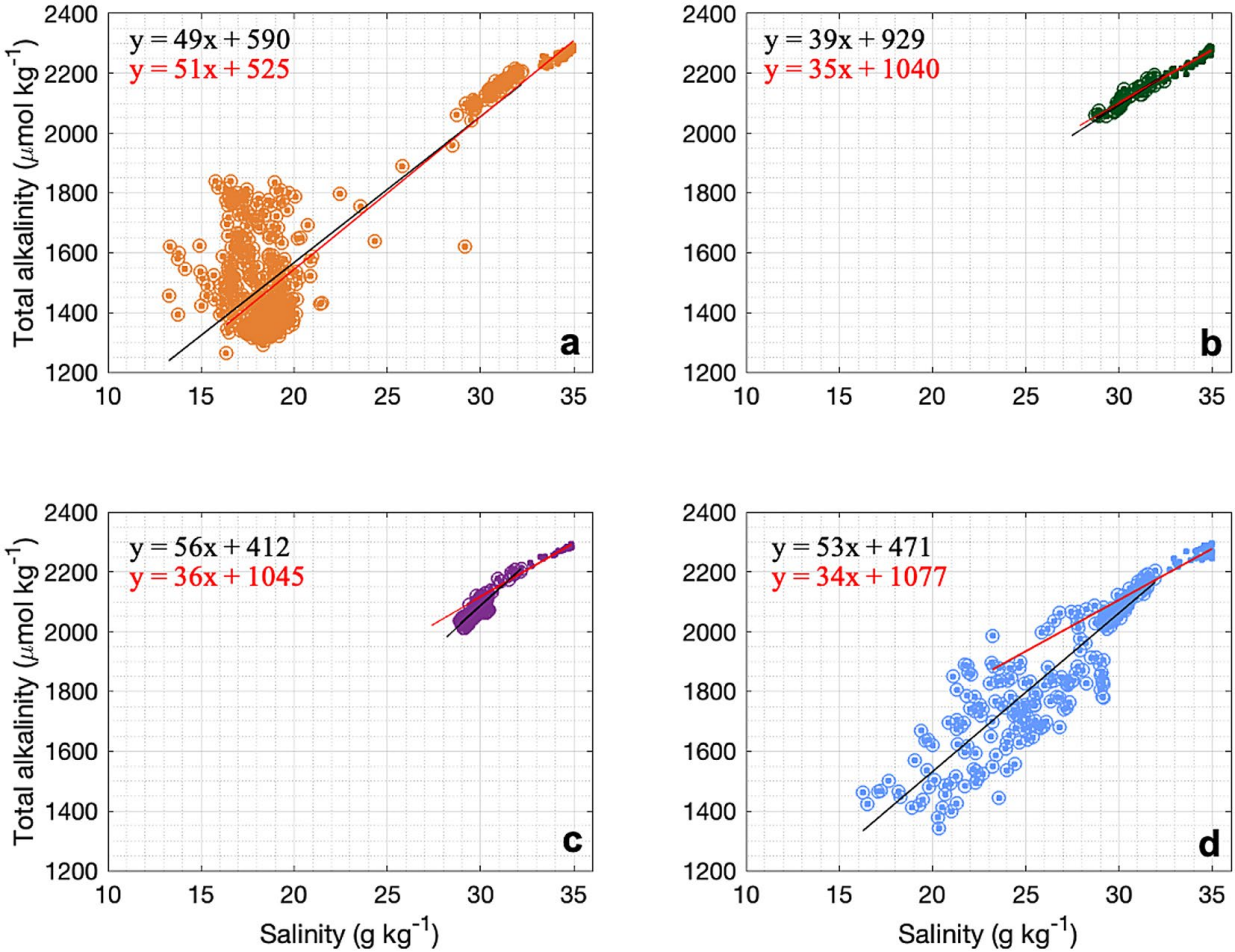
Relationships between TA and salinity for underway measurements and CTD-rosette water samples in Sherard Osborn Fjord (**a**), Petermann Fjord (**b**), the Nares Strait (**c**) and the Lincoln Sea (**d**). Black lines indicate linear regression for the data with salinity lower than 32.5 (circles) and red lines indicate linear regression for the whole water column. All linear regressions have coefficient correlation between 0.7 and 1 with p-value less than 0.05.

Discrete seawater pH was determined on the total scale using a spectrophotometric method using purified m-Cresol Purple purchased from Dr. Eric Achterberg, GEOMAR, Kiel, Germany (mCP,^45,46^) The indicator was adjusted to a pH in the same range as the samples, approximately ± 0.2 pH units, by adding a small volume of concentrated HCl or NaOH. Before running a set of samples, the pH of the indicator was measured using a 0.02 cm cuvette. Measurements were performed on board within hours of sampling and samples were thermostated to $25\,^{\circ }\hbox {C}$ in a water bath 30 min prior to analysis. An automatic system^47^ was used in which the sample and indicator were mixed in a syringe (Kloehn) before being injected into a 1 cm cuvette of a diode array spectrophotometer (Agilent 8453), where the absorbance was measured at wavelengths 434, 578, and 730 nm, the latter accounting for background absorbance. The influence of indicator additions on the seawater pH samples was corrected^48^. The pH values were corrected to $25\,^{\circ }\hbox {C}$ on the total scale. The accuracy was determined by the pureness of the indicator and by analyzing certified reference material (CRM batch *#181*). The latter measurements indicated that accuracy should be well below 0.01 pH unit. The precision as determined by replicates from the same sample bottle was in the range of ± 0.001 pH unit. Discrete TA was determined using a semi-open cell potentiometric titration method using a 5-point Gran evaluation^49^. The system measures alkalinity in $\mu$mol $\hbox {L}^{-1}$ using a nominal hydrochloric acid (HCl) concentration of 0.05 mol $\hbox {L}^{-1}$ and 0.65 mol $\hbox {L}^{-1}$ sodium chloride (NaCl). Certified reference material (CRM Batch *#181*) was used to determined accuracy. For all samples and CRM analyses, the alkalinity in $\mu$mol $\hbox {kg}^{-1}$ was calculated using the salinity (from the CTD bottle file and the certified salinity, respectively) and the temperature measured at the beginning of the titration. The sample results were then multiplied by the factor determined from the CRM measurements at each individual station, and the correction was always below 0.5 %. For discrete TA measurements, the mean ± standard deviation of absolute differences for duplicates CRM/samples was 2.6 ± 2.2 $\mu$mol $\hbox {kg}^{-1}$, based on the absolute differences of CRM duplicates (n = 25) and 3.1 ± 2.8 $\mu$mol $\hbox {kg}^{-1}$ based on the absolute differences of seawater sample duplicates (n = 66). For discrete pH measurements, the mean ± standard deviation absolute difference was 0.0023 ± 0.002 pH units, based on the absolute differences of seawater sample duplicates (n = 393).

### Physical and biogeochemical properties

The spatial distribution of surface water parameters between August 7$^{th}$ and September 7$^{th}$ reveal distinct patterns. The surface conservative temperature (hereafter temperature) ranged from -0.98 to 4.04 °C, with an average of 085 ± 1.7 °C (Fig. 2a), while surface absolute salinity (hereafter salinity) showed variations between 13.21 g $\hbox {kg}^{-1}$ and 30.54 g $\hbox {kg}^{-1}$, averaging 23.78 ± 5.6 g $\hbox {kg}^{-1}$ (Fig. 2b). Surface chlorophyll-a (hereafter *chl-a*) spanned from 0.19 to 4.63 RFU (relative fluorescent unit), with a mean of 0.51 ± 0.55 RFU (Fig. 2c). Spatially, the mean surface temperature in the Nares Strait, Petermann Fjord, and the Lincoln Sea was about $3\,^{\circ }\hbox {C}$ lower compared to the mean surface temperature observed in Sherard Osborn Fjord (Fig. 2a, Supplementary Table 1), gradually increasing toward the latter. In contrast, the spatial pattern of surface salinity lacked the systematic nature observed in surface temperature (Fig. 2b) with no significant correlation between these two parameters in the Lincoln Sea and Petermann Fjord and with a weak correlation in the Nares Strait and Sherard Osborn Fjord (Supplementary Figure 2). Surface *chl-a* was generally low ($< 1.5$ RFU) in both fjords, while in the Nares Strait and in the Lincoln Sea it was higher ($> 2.0$ RFU) (Fig. 2c, Supplementary Table 1). During the cruise, the mean $\hbox {pCO}_{2}^{sw}$ was about 260 ± 32 $\mu$atm with an average uncertainty of 7 $\mu$atm and the air partial pressure of $\hbox {CO}_{2}$ ($\hbox {pCO}_{2}^{atm}$) was 404 ± 3 $\mu$atm (Fig. 2d), indicating an overall surface water undersaturation of $\hbox {CO}_{2}$ compared to the atmosphere. The mean values of $\hbox {pCO}_{2}^{sw}$ were 278 ± 20 $\mu$atm, 260 ± 23 $\mu$atm, 284 ± 5 $\mu$atm, and 257 ± 32 $\mu$atm in the Nares Strait, the Lincoln Sea, Petermann Fjord, and Sherard Osborn Fjord, respectively (Supplementary Table 1). Furthermore, significant variations in $\hbox {pCO}_{2}^{sw}$ were found locally in each area and particularly in Sherard Osborn Fjord (Fig. 2d). $\hbox {pCO}_{2}^{sw}$ was generally lower in Sherard Osborn Fjord, with the lowest values observed in the surface layer at its entrance, and in the Lincoln Sea (area east of $57^{\circ }\hbox {W}$) notably dropping to 189 $\mu$atm at some locations (Fig. 2d). Comparably high $\hbox {pCO}_{2}^{sw}$ was observed in the inner part of Sherard Osborn Fjord with values ranging between 300 and 380 $\mu$atm, in contrast to the general low $\hbox {pCO}_{2}^{sw}$ in the rest of the fjord, as well as of the entire region covered by the expedition. The maximum $\hbox {pCO}_{2}^{sw}$ values, approximately 380 $\mu$atm, were recorded in an area with large horizontal variability close to the ice-tongue margin of Ryder Glacier (Fig. 2d). The low $\hbox {pCO}_{2}^{sw}$ at the entrance of Sherard Osborn Fjord is associated with colder and fresher surface waters (Fig. 2a-b, d). In contrast, $\hbox {pCO}_{2}^{sw}$ levels in Petermann Fjord showed relatively smaller variations compared to those in Sherard Osborn Fjord (Fig. 2d).

In polar oceans, winter sea-ice formation and air-sea heat exchange deepen the mixed layer by increasing surface salinity and cooling. In summer, warming and freshwater inputs drive upper ocean stratification, forming a pycnocline that isolates the cooler winter water below. This creates a temperature minimum layer (TML), where winter water is colder than both the summer surface and deeper layers^50^. In the collected summertime temperature profiles, we assume that the TML water is representative of surface conditions in late winter just before re-stratification occurs. Furthermore, following the approach of refs.^51,52^, we use this TML as a proxy to identify the biogeochemical properties of the waters that were last in contact with the atmosphere during the previous winter (Supplementary Table 1). Of the four areas where the water column was sampled, the Lincoln Sea and Sherard Osborn Fjord exhibited TML with water temperatures below $-1.5\,^{\circ }\hbox {C}$ (Fig. 3a), suggesting intensive sea-ice formation during the previous winter. The average TML dissolved inorganic carbon (DIC) content of 2087 ± 12 $\mu$mol $\hbox {kg}^{-1}$ and 2075 ± 14 $\mu$mol $\hbox {kg}^{-1}$, and average total alkalinity (TA) content of 2180 ± 10 $\mu$mol $\hbox {kg}^{-1}$ and 2173 ± 11 $\mu$mol $\hbox {kg}^{-1}$ in Sherard Osborn Fjord and Petermann Fjord, respectively, indicated similar carbonate properties of the TML waters in the two fjords (Fig. 3c-e, h-j). Consistently in Sherard Osborn Fjord, low $\hbox {pCO}_{2}^{sw}$ values ($< 300$ $\mu$atm Fig. 3e) were observed throughout the first meters of the surface water column ($\sim$20 m depth), coinciding with DIC content lower than 2000 $\mu$mol/kg (Fig. 3d). Below the shallow layer of low salinity, the DIC content and $\hbox {pCO}_{2}^{sw}$ values increased substantially with depth, reaching their maximum levels at approximately 50 m depth in Sherard Osborn and Petermann fjords, and 70 m depth in Nares Strait and Lincoln Sea.

**Fig. 5:**
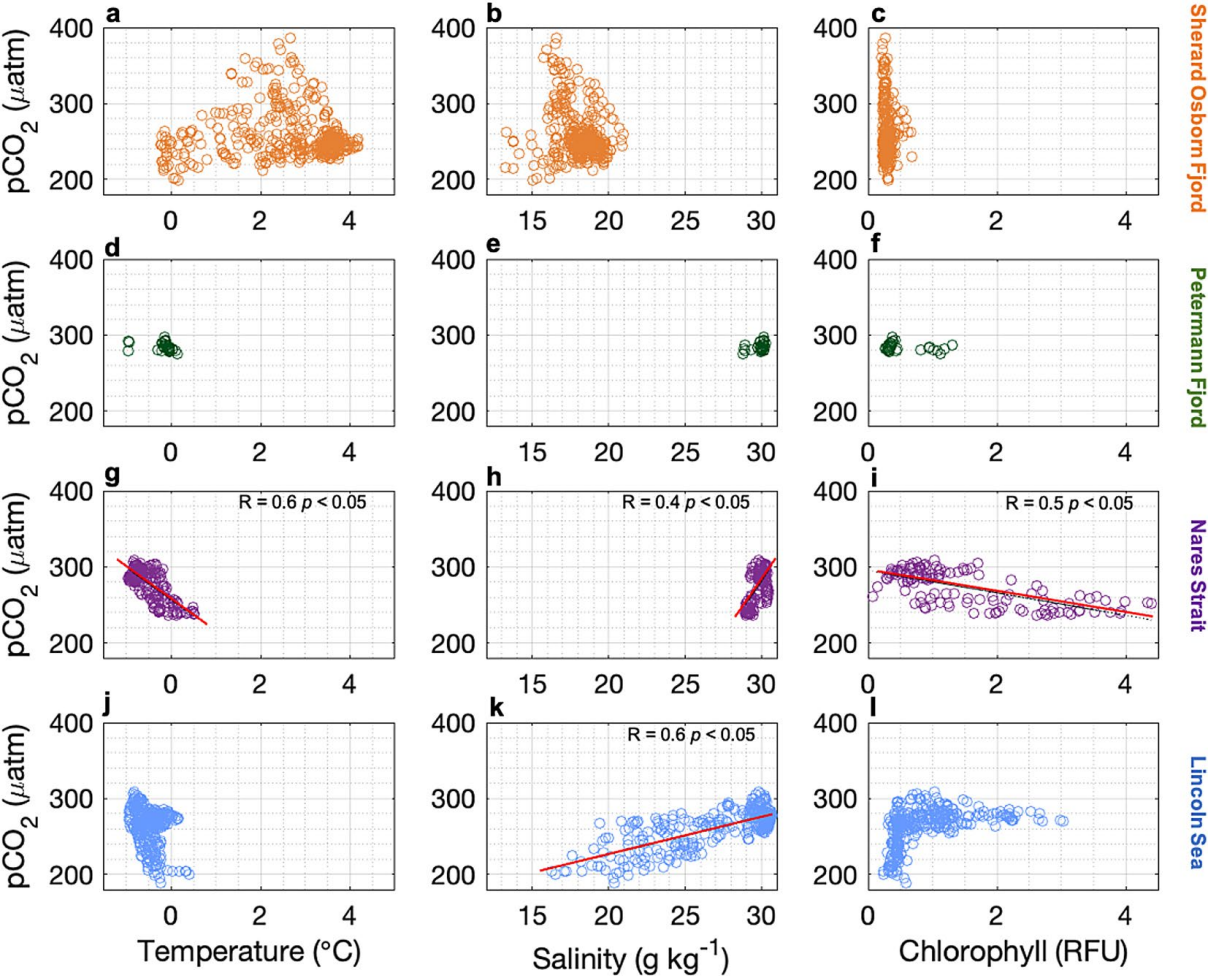
Relationships between surface seawater temperature, salinity and chlorophyll a with $\hbox {pCO}_{2}$ in Sherard Osborn Fjord (orange dots), Petermann Fjord (green dots), Nares Strait (purple dots) and Lincoln Sea (blue dots). The dash lines are regression lines with *p* $< 0.05$.

**Fig. 6:**
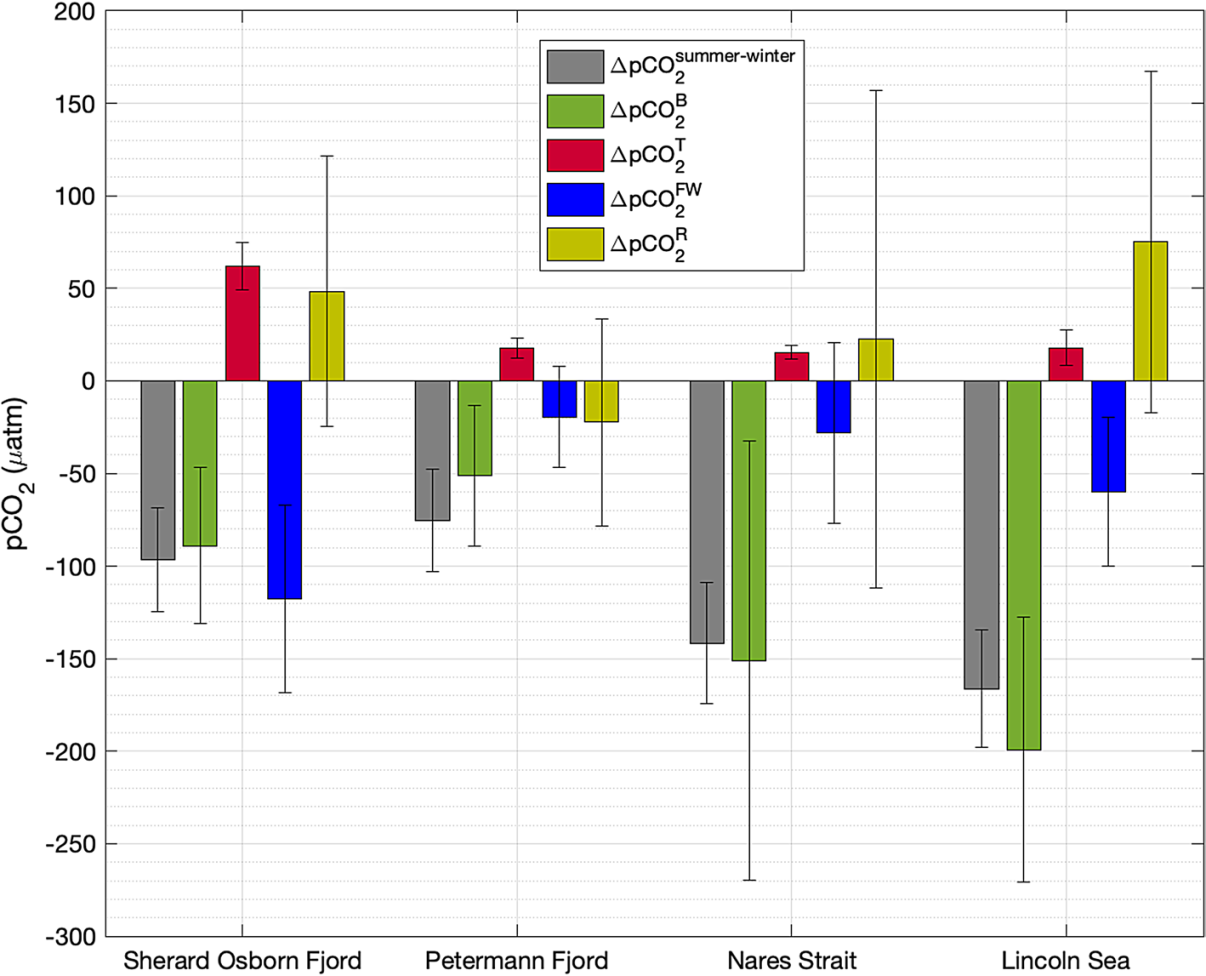
Total seasonal changes in surface $\hbox {pCO}_{2}$ either observed (grey) or estimated from change in biological activity (green), thermal effect (red), freshwater inputs (blue), and the residuals (khaki) including air-sea $\hbox {CO}_{2}$ flux and physical ocean processes. The standard deviations of the propagation of uncertainties exercise are shown by the black error bars.

**Fig. 7:**
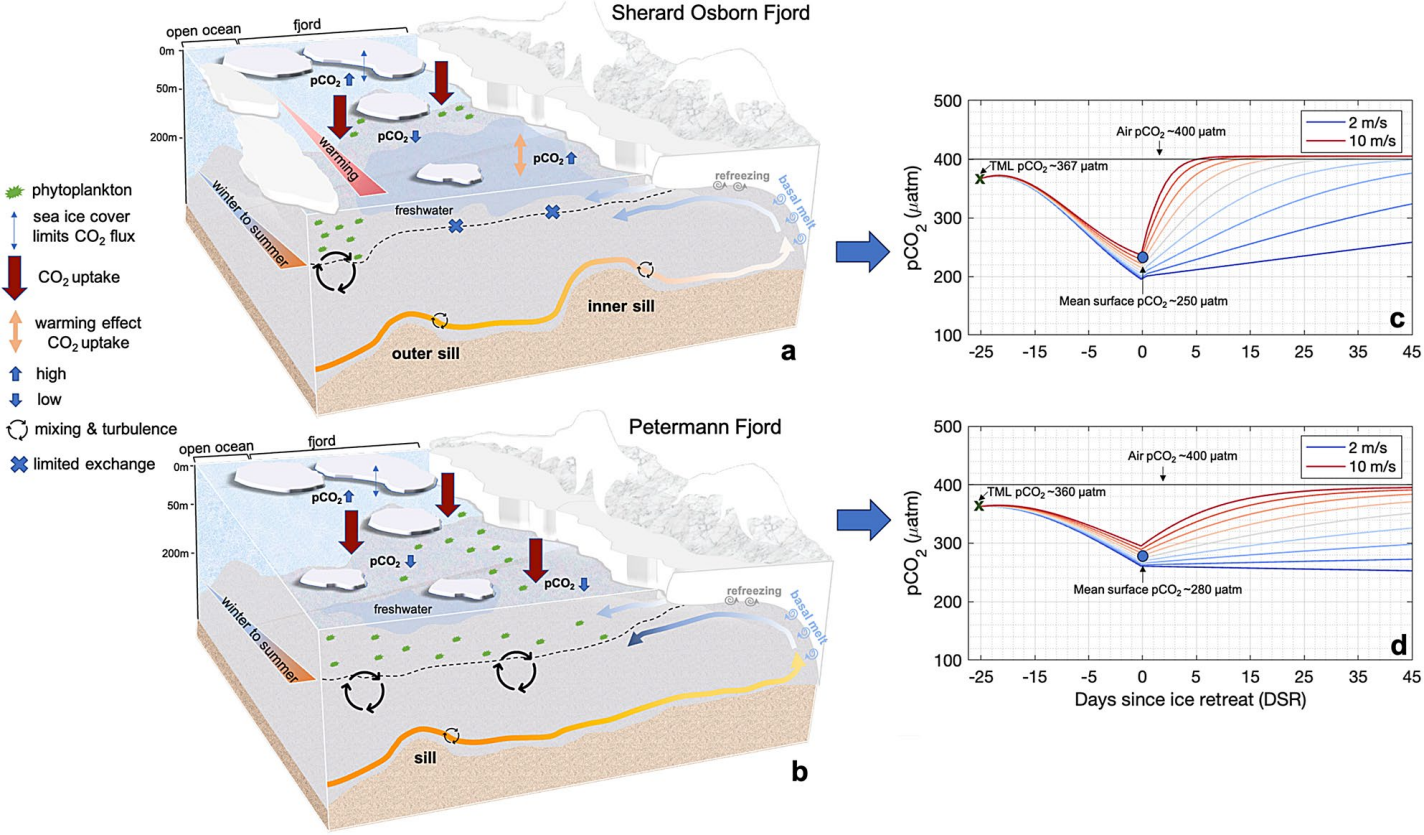
Conceptual diagrams of the fjord surface water $\hbox {pCO}_{2}$ evolution and its drivers in Sherard Osborn Fjord (**a**) and Petermann Fjord (**b**). Local variation on a fjord scale is mainly controlled by the freshwater inputs and the subsequent high stratification as shown by the results of a number of 1D dynamic simulations (**c**-**d**). Modeled $\hbox {pCO}_{2}$ (curves with the color matching the wind speed ranging between 2 m $\hbox {s}^{-1}$ and 10 m $\hbox {s}^{-1}$) with initial values (black crosses) and model settings derived from the average observations at the temperature minimum layer (TML) in Sherard Osborn Fjord (**c**) and Petermann Fjord (**d**). Days since ice retreat (DSR) for $\hbox {pCO}_{2}$ simulations were negative before the sampling time (DSR = 0) corresponding to the period of sea-ice melt and positive after the sampling time corresponding the period of post sea-ice melt. The horizontal line is the atmospheric $\hbox {pCO}_{2}$.

The alkalinity of the ocean evolves in tandem with the variations in salinity, influenced by freshwater inputs and water mixing processes. Consequently, the pattern of surface water distribution of TA aligns with variations in salinity in the polar oceans^53^. This relationship enables the estimation of TA values at salinity levels where direct measurements may be lacking, such as in the freshwater end-members (Fig. 4). Surface and subsurface waters with salinity up to 32.5 g $\hbox {kg}^{-1}$ in Sherard Osborn Fjord showed low zero-salinity TA end-member (TA0) of 590 $\mu$mol $\hbox {kg}^{-1}$ (Fig. 4a), while the remaining areas in the Nares Strait and the Lincoln Sea had a lower TA0 of around 412 $\mu$mol $\hbox {kg}^{-1}$ and 471 $\mu$mol $\hbox {kg}^{-1}$, respectively. In Petermann Fjord, the TA:Salinity relationship indicates conservative mixing with a higher alkalinity end-member (Fig. 4b, TA0 at 929 $\mu$mol $\hbox {kg}^{-1}$). Furthermore, in deeper waters with salinity higher than 32.5 g $\hbox {kg}^{-1}$, mixing appears to be non-conservative, with TA contents varying by around 100 $\mu$mol $\hbox {kg}^{-1}$ for minor variations in salinity of $\sim$2 g $\hbox {kg}^{-1}$ (Fig. 4) and indicates that other processes, such as calcium carbonate formation and dissolution, and horizontal advection, drive these variations in TA at depth. The different mixing regimes for TA in the surface waters of the region indicate control by different processes (Fig. 4); the low TA end-member is consistent with the high influence of sea-ice melt (50–400 $\mu$mol $\hbox {kg}^{-1}$,^54,55^), and glacial meltwater (100-400 $\mu$mol $\hbox {kg}^{-1}$,^55,56^) while higher TA end-member might reflect inputs from pan-Arctic rivers with elevated TA (1000-1500 $\mu$mol $\hbox {kg}^{-1}$,^57–60^); Arctic rivers runoff being carried by the Transpolar Drift across the Central Arctic and out through the Nares Strait^23,61,62^, contributing approximately 8–10 % of the water mass composition north of the strait^23^.

### Air-Sea $\hbox {CO}_{2}$ flux

Based on the difference between $\hbox {pCO}_{2}^{sw}$ and $\hbox {pCO}_{2}^{atm}$, and wind speeds measured during the cruise, the estimated air-sea $\hbox {CO}_{2}$ flux, $\hbox {F}_{CO_{2}}$ (mmol $\hbox {m}^{-2}$ $\hbox {d}^{-1}$), was calculated according to Eq. 1:

1 $$\begin{aligned} F_{CO_{2}} = kK_{0} (pCO_{2}^{sw} - pCO_{2}^{atm}), \end{aligned}$$

where, k (m $\hbox {d}^{-1}$) is the gas transfer velocity calculated according to ref.^63^ using 0.251 for the empirical coefficient correcting for the gas exchange-wind speed relationship, the dimensionless Schmidt number at measured temperature and salinity, and the concurrent wind speed. $\hbox {K}_{0}$ is the solubility according to ref.^64^ and using surface temperature and surface salinity. A positive flux indicates $\hbox {CO}_{2}$ outgassing from the ocean to the atmosphere, and a negative flux indicates ocean uptake. The uncertainty in $\hbox {F}_{CO_{2}}$ arises from several factors, including biases related to (i) surface waters influenced by sea-ice cover, (ii) uncertainty in wind speed, (iii) calculated uncertainty in $\hbox {pCO}_{2}^{sw}$, and (iv) uncertainty in gas exchange parameterizations themselves^65,66^. Although different from open ocean conditions, our study area is characterized by vast fjords with low ice coverage during sampling, we have therefore used the parameterization outlined by ref.^63^. Furthermore, accounting for the significant error in the Schmidt number, constituting the primary source of uncertainty in flux calculations according to ref.^63^, we acknowledge the flux calculation uncertainty to be 20 % as previous methodologies scaling with sea-ice coverage overestimate $\hbox {CO}_{2}$ fluxes^67,68^. Therefore, our focus covers a wide range of potential $\hbox {F}_{CO_{2}}$ values that could occur in the region (Supplementary Figure 3). The air-sea $\hbox {CO}_{2}$ flux ranged from -186 and -0.01 mmol $\hbox {m}^{-2}$ $\hbox {d}^{-1}$ (Supplementary Figure 3), with an average uncertainty of 8 mmol $\hbox {m}^{-2}$ $\hbox {d}^{-1}$ indicating that seawater acted as a sink for atmospheric $\hbox {CO}_{2}$ in the northwestern Greenland region during the expedition. Maximum uptake was observed at the Sherard Osborn Fjord entrance and the Nares Strait and was associated with higher wind speeds (Supplementary Figure 4).

### Determination of physical and biogeochemical drivers to $\hbox {pCO}_{2}^{sw}$ changes

The mechanisms controlling ocean $\hbox {pCO}_{2}$ can be translated into the following equation Eq. 2:

2 $$\begin{aligned} \Delta pCO_{2}^{summer-winter} \approx \Delta pCO_{2}^{T} + \Delta pCO_{2}^{B} + \Delta pCO_{2}^{FW} + \Delta pCO_{2}^{R}, \end{aligned}$$

where the superscripts correspond to the different contributions; B denotes the biological effect, T denotes the thermal effect, FW denotes the effect of freshwater inputs, and R represents the residuals, including air-sea $\hbox {CO}_{2}$ flux and ocean circulation.

The thermal effect on $\hbox {pCO}_{2}^{sw}$ ($\Delta pCO_{2}^{T}$) was calculated using simulated changes in surface temperature from the onset of sea-ice melting to the time of sampling. Changes in surface temperature were simulated from the mean temperature of the TML to the observed summer surface temperatures for each station, assuming a linear increase. Specifically, for the Lincoln Sea, and Sherard Osborn and Petermann fjords, surface temperatures were simulated from July 15$^{th}$ (based on sea-ice Terra MODIS imagery from NASA WorldView) to August 7$^{th}$, and for the Nares Strait from June 15$^{th}$ to August 7$^{th}$. $\hbox {pCO}_{2}^{sw}$ dependence on surface temperature was determined by comparing $\hbox {pCO}_{2}^{sw}$ at the TML with the observed $\hbox {pCO}_{2}^{sw}$ at each station: we calculated the relationship daily and summed it over the onset of the sea-ice melting-to-summer period Eq. 3:

3 $$\begin{aligned} \Delta pCO_{2}^{T} = \sum _{i=1}^n \partial _{t}pCO_{2}^{T}(i), \end{aligned}$$

where $\partial _{t}pCO_{2}^{T}$ was determined from Eq. 4^69^:

4 $$\begin{aligned} \partial _{t}pCO_{2}^{T}(i) = pCO_{2}^{sw}(t_{i}) - pCO_{2}^{sw}(t_{i-1}), \end{aligned}$$

The $\hbox {pCO}_{2}^{sw}$ value corresponding to a simulated temperature was determined as follows (Eq. 5):

5 $$\begin{aligned} pCO_{2}^{sw}(t_{i}) = pCO_{2}^{sw}(t_{i-1}) \times e^{0.0423 (Tt_{i} - Tt_{i-1})}, \end{aligned}$$

In Eq. 4 and Eq. 5*t* is time and *T* is the temperature of the ocean surface simulated during *n* days with a resolution of 1 day.

The biological impact on $\hbox {pCO}_{2}^{sw}$ ($\Delta pCO_{2}^{B}$) was calculated from Eq. 6^51^:

6 $$\begin{aligned} \Delta pCO_{2}^{B} = -\beta \times pCO_{2}^{TML} \times \frac{NCP}{TnDIC}, \end{aligned}$$

where -$\beta$ is the Reveller buffer factor calculated from the CO2SYS program and T*n*DIC is the integrated amount of normalized DIC between the surface and the TML. The difference between the preformed winter DIC content and the measured DIC content integrated within the summer surface waters is assumed to represent the seasonal biological drawdown of DIC, that is, the net community production (NCP in mol C $\hbox {m}^{-2}$), estimated from Eq. 7:

7 $$\begin{aligned} NCP = -\int _{0}^{Z_{TML}} (nDIC_{Z} - nDIC_{TML})dx, \end{aligned}$$

where $\hbox {Z}_{Tmin}$ is the depth of the core of the TML, *n*$\hbox {DIC}_{Z}$ is the value of normalized DIC at depth z, *n*$\hbox {DIC}_{TML}$ is the value of normalized DIC in the TML. To correct for the effect of dilution on DIC and TA due to freshwater inputs during the onset of sea-ice melting-to-summer transition, DIC and TA were normalized to the mean salinity of each profile (noted as *n*DIC and *n*TA). Based on the regression line calculated for each area and using all data from the water column (red regression lines in Fig. 5) in their respective TA:Salinity and DIC:Salinity relationships, we estimated their respective end-members at salinity 0 for TA and DIC. Values for *n*TA and *n*DIC were then calculated using the normalization from ref.^70^, Eq. 8:

8 $$\begin{aligned} nX = \frac{X_{obs} - X_{FW}}{S_{obs}} \times S_{TML} + X_{FW}, \end{aligned}$$

where *n*X is the normalized DIC or TA. *X*$_{obs}$ and *X*$_{FW}$ are the DIC or TA of the observed seawater end-member and estimated freshwater end-member. *S*$_{obs}$ and *S*$_{TML}$ are the observed salinity and the salinity in the TML, respectively.

Freshwater inputs $\Delta pCO_{2}^{FW}$ is estimated by Eq. 9:

9 $$\begin{aligned} \Delta pCO_{2}^{FW} = pCO_{2}^{sw-est} - pCO_{2}^{sw-obs}, \end{aligned}$$

where $\hbox {pCO}_{2}^{sw-obs}$ corresponds to the surface water $\hbox {pCO}_{2}$ data collected during the cruise. $\hbox {pCO}_{2}^{sw-est}$ corresponds to the surface water $\hbox {pCO}_{2}$ recalculated by the CO2SYS program using estimated TA, DIC and salinity influenced only by freshwater inputs in surface waters. At each station, the surface freshwater fraction was calculated as follows, Eq. 10:

10 $$\begin{aligned} fFW = 1 - \frac{S_{obs}}{S_{TML}}, \end{aligned}$$

The estimated summer surface water parameters only influenced by the addition of freshwater to the initial winter surface water conditions are calculated as follows, Eq. 11:

11 $$\begin{aligned} X_{est} = X_{FW} \times fFW + X_{TML} \times (1 - fFW), \end{aligned}$$

where $\hbox {X}_{est}$ corresponds to TA, DIC and salinity of surface water, $\hbox {X}_{FW}$ corresponds to the TA, DIC, and salinity characteristics of freshwater sources, estimated using linear regressions and their associated errors in TA:Salinity and DIC:Salinity relationships, and $\hbox {X}_{TML}$ corresponds to the TA, DIC, and salinity properties of the TML for each station.

Uncertainties may arise from the propagation of errors from the initial measured or estimated parameters, as well as intrinsic errors related to the method chosen to address the contribution of each driving factor of $\hbox {pCO}_{2}^{sw}$ change, through the equations above. To investigate the uncertainties, we conducted Monte-Carlo experiments involving 1000 repetitions of Eqs. 3, 6 and 9 resolutions. Random noise was added to the parameters within their respective error limits (Supplementary Table 1). Subsequently, the uncertainties associated with each contribution ($\Delta pCO_{2}^{T}$, $\Delta pCO_{2}^{B}$, and $\Delta pCO_{2}^{FW}$) were derived from the standard deviation of the experiment.

## Results

To explore the processes that influence surface water $\hbox {pCO}_{2}$ (hereafter $\hbox {pCO}_{2}^{sw}$), we examine high-resolution measurements (Fig. 1b) of surface properties such as salinity, temperature, and *chl-a* during late summer 2019. Subsequently, the relationships between these properties and $\hbox {pCO}_{2}^{sw}$ are analyzed. Next, we decompose the changes in $\hbox {pCO}_{2}^{sw}$ from the initial stages of sea-ice retreat–when the waters reflect the characteristics of winter surface water–to the sampling period–when the waters exhibit summer surface water conditions. This decomposition involves contributions of thermal, freshwater inputs and biological effects through the analysis of vertical profiles. Specifically, we denote the differences between the observed $\hbox {pCO}_{2}^{sw}$ (representative of summer surface waters) and the observed $\hbox {pCO}_{2}$ in the subsurface below the mixed-layer (representative of previous winter surface waters) using data collected from water column profiles. This approach allows us to estimate the relative contribution of each driver controlling $\hbox {pCO}_{2}^{sw}$, from the onset of sea-ice melting to late summer conditions.

We first analyzed the distribution of $\hbox {pCO}_{2}^{sw}$ in relation to surface *chl-a*, temperature, and salinity using simple linear regressions for each region. The correlations were evaluated at the confidence level 95%, with Fig. 5 only including those with *p* $< 0.05$. Overall, $\hbox {pCO}_{2}^{sw}$ showed no strong correlations with temperature, salinity, or *chl-a* (Fig. 5). In Nares Strait, $\hbox {pCO}_{2}^{sw}$ exhibited an increasing trend with salinity (Fig. 5h) and a negative relationship with temperature (Fig. 5g) and *chl-a* (Fig. 5i). In the Lincoln Sea, $\hbox {pCO}_{2}^{sw}$ also correlated positively with salinity (Fig. 5k), while its relationship with temperature or *chl-a* show no significant correlation (Fig. 5k-l). In contrast, no significant correlations were found in Petermann Fjord (Fig. 5d-f) and Sherard Osborn Fjord (Fig. 5a-c). In general, the absence of strong correlations among key variables likely reflects the limited duration of sampling and the complex interplay of biological activity, temperature, and freshwater inputs, making it challenging to disentangle their individual effects. To elucidate these dynamics, we further quantify the relative contributions of each factor driving $\hbox {pCO}_{2}^{sw}$ variability in the region.

The seasonal evolution of $\hbox {pCO}_{2}^{sw}$ ($\Delta pCO_{2}^{summer-winter}$) in the four areas, indicative of $\hbox {pCO}_{2}^{sw}$ variations between the onset of sea-ice melting and late summer, was estimated by the difference between the observed $\hbox {pCO}_{2}^{sw}$ (summer surface waters) and the observed $\hbox {pCO}_{2}$ at the TML (winter surface waters) at each station. In the TML, $\hbox {pCO}_{2}$ ranged between 347 and 637 $\mu$atm in the region (Supplementary Table 1). In the Lincoln Sea, seasonal variations (or differences between $\hbox {pCO}_{2}^{sw}$ and $\hbox {pCO}_{2}$ at TML) reaching values as substantial as -320 $\mu$atm were observed. In particular, these elevated values can also be influenced by subsurface Pacific-origin waters^71,72^, which are characterized by high $\hbox {CO}_{2}$ concentrations^73^. Fig. 6 illustrates the intricate interplay of these four summed internal processes (thermal, biological, freshwater inputs and residuals) influencing seasonal $\hbox {pCO}_{2}^{sw}$ changes between the onset of sea-ice melting and the late summer survey, synergistically or in opposition.

The regional distribution of $\Delta pCO_{2}^{T}$, illustrated in Supplementary Figure 5, reveals that the Nares Strait experiences minimal temperature changes, resulting in negligible thermal effects on $\hbox {pCO}_{2}^{sw}$. The Lincoln Sea shows minor thermal effects, while Sherard Osborn Fjord in general exhibits large thermal influences exceeding 20 $\mu$atm, with the most substantial effects, up to 97 $\mu$atm, observed in the inner part of the fjord. On average, considerable biological effects on $\hbox {pCO}_{2}^{sw}$ were estimated in the Nares Strait and the Lincoln Sea (Fig. 6). In particular, the influence was particularly pronounced in the Lincoln Sea, where estimated values of $\Delta pCO_{2}^{B}$ reached -550 $\mu$atm (Supplementary Fig. 5). In Sherard Osborn and Petermann fjords, the contribution varied significantly, ranging from -212 to -10 $\mu$atm and from −114 to −3 $\mu$atm, respectively (Supplementary Figure 5). In contrast, Sherard Osborn Fjord showed a moderate to large biological effect, and Petermann Fjord exhibited a lower biological effect on average (Fig. 6). Locally strong biological effects, such as those observed in front of the Ryder glacier (Supplementary Fig. 5), were observed despite the fact that fluorescence levels were consistently low during the sampling period (Supplementary Fig. 6). The regional influence of freshwater inputs on $\hbox {pCO}_{2}^{sw}$ is illustrated in Supplementary Figure 5, which shows, as expected, high $\Delta pCO_{2}^{FW}$ values in Sherard Osborn Fjord, with reductions of up to -177 $\mu$atm, particularly near the Ryder Glacier. While $\hbox {pCO}_{2}^{sw}$ in Nares Strait and the Lincoln Sea is moderately affected by freshwater inputs, Petermann Fjord exhibits the weakest influence.

We finally defined the effect of residuals on seasonal $\hbox {pCO}_{2}^{sw}$ changes as the sum of $\Delta pCO_{2}^{T}$, $\Delta pCO_{2}^{B}$, and $\Delta pCO_{2}^{FW}$ minus $\Delta pCO_{2}^{summer-winter}$, denoted as $\Delta pCO_{2}^{R}$. Upon removing the biological, thermal and freshwater components, the residual effects on $\hbox {pCO}_{2}^{sw}$ can be attributed to air-sea $\hbox {CO}_{2}$ fluxes and physical processes such as advection, vertical mixing and variability of water masses. The residual contributions are notably high in the Lincoln Sea and in Sherard Osborn Fjord, exceeding 100 $\mu$atm close to the Ryder Glacier (Supplementary Figure 5). They were slightly positive in the Nares Strait and mainly negative in Petermann Fjord, with one station showing positive values in the latter. These contributions varied greatly, ranging between -85 to 282 $\mu$atm, indicating that these large values could not be solely explained by gas exchange with the atmosphere and lateral/vertical mixing of water masses, they may also be attributed to calculation uncertainties.

To investigate differences in $\hbox {pCO}_2$ dynamics between Petermann and Sherard Osborn fjords we apply here a 1D dynamic model, based on the concept of days since ice retreat (DSR), to simulate $\hbox {pCO}_2^{sw}$ evolution in both systems. The absence of sea-ice exposes surface waters directly to atmospheric $\hbox {CO}_{2}$ flux, increasing $\hbox {pCO}_{2}^{sw}$. This suggests that $\hbox {pCO}_{2}^{sw}$ is not solely dependent on sea-ice cover but also on the duration of open water^74^. The purpose of the following simulations, admittedly highly simplified, is to evaluate the difference between a system with a shallow, strongly stratified mixed-layer (Fig. 7a, Sherard Osborn Fjord) and the surface waters more commonly observed in Greenlandic fjords during summer (Fig. 7b, Petermann Fjord). The initial values used in the simulations are derived from observations in summer 2019, making difficult to extrapolate the results to other seasons and years. Additionally, since the simulations employ linear parameter changes, it can only project mean changes, not the time-varying rates of warming/cooling and freshwater inputs for instance.

We define the day of ice retreat as the day when sea-ice concentration substantially decreases, based on Terra MODIS imagery from NASA WorldView. Positive or negative DSR values indicate two periods: a sea-ice melt period ($\hbox {DSR} < 0$) and a post sea-ice melt period ($\hbox {DSR} > 0$). Before sea-ice begins to melt, it is assumed that $\hbox {pCO}_{2}^{sw}$ is similar to the values measured in the TML, representing the winter surface water conditions. Therefore, the initial surface temperature, salinity and $\hbox {pCO}_{2}^{sw}$ are set based on the mean value of all profiles in the TML for each fjord (supplementary Table 1). During the sea-ice melt period ($\hbox {DSR} < 0$), we assume that the surface temperature linearly increases to the maximum surface temperature observed over each fjord, $+6\,^{\circ }\hbox {C}$ and $+1\,^{\circ }\hbox {C}$ for 25 days in Sherard Osborn and Petermann fjords, respectively, while the surface salinity linearly decreases to their observed means at $\hbox {DSR} = 0$. At $\hbox {DSR} = 0$, surface temperature, surface salinity and surface TA are set to the values observed at the sampling time, and during the ice-free period ($\hbox {DSR} > 0$) these parameters remain constant. Heating, melting, and surface stratification occurred before $\hbox {DSR} = 0$ and remain consistent thereafter during the post-sea-ice melt stage. We assume linear freshwater inputs at $\hbox {DSR} < 0$ at a rate of 0.3 % and 0.1 % melt per day for Sherard Osborn and Petermann fjords, respectively. Net community production (hereafter NCP) is estimated during the growth period from the onset of sea-ice melt to summer (Eq. 7) and we assume a linear biological $\hbox {CO}_{2}$ uptake occurred before $\hbox {DSR} = 0$ only in Sherard Osborn Fjord (1.3 mol C $\hbox {m}^{-2}$), whereas in Petermann Fjord, linear biological $\hbox {CO}_{2}$ uptake occurred before and after $\hbox {DSR} = 0$ (0.6 mol C $\hbox {m}^{-2}$). In addition, the mixed-layer depths are assumed to shallow linearly at a rate of 0.5 m and 0.3 m per day from the TML depth (43 m and 33 m for Sherard Osborn and Petermann fjords, respectively) to the summer mixed-layer depth (6 m and 12 m for Sherard Osborn and Petermann fjords, respectively, based on density differences of 0.125 kg $\hbox {m}^{-3}$ from the shallowest measured value) for the period $\hbox {DSR} < 0$. A range of wind speeds between 2 and 10 m $\hbox {s}^{-1}$ is considered for air-sea $\hbox {CO}_{2}$ flux estimation, impacting simulated $\hbox {pCO}_{2}^{sw}$.

At the onset of the sea-ice melt period, biological carbon uptake driven by primary production may leads to a reduction in DIC and, consequently, $\hbox {pCO}_{2}^{sw}$ in the surface waters decreases in both fjords^30^ (Fig. 7c-d). During this brief spring bloom period, the influence of freshwater inputs (i.e., sea-ice melt and glacial meltwater) on the fjord systems remains low, and the upper water column is assumed to be well mixed leading to relatively homogeneous $\hbox {CO}_{2}$ undersaturation^30^. As the melt season progresses (toward DSR = 0), freshwater inputs act to dilute $\hbox {pCO}_{2}^{sw}$, as evidenced by the modeled $\hbox {pCO}_{2}$ at DSR = 0, with the combined effects of primary production (Fig. 7c-d). In contrast to Sherard Osborn Fjord, the weaker upper-ocean stratification in Petermann Fjord is likely explain by its hydrographic connectivity with the Nares Strait and, the timing of the cruise which took place in early fall (DSR = 0). By this time, freshwater inputs influence was less pronounced, certainly flushed out of the fjord, and the upper-water column remained well mixed compared to Sherard Osborn Fjord (Fig. 7a-b). At this stage and in the following days, primary production continued to counteract $\hbox {CO}_{2}$ influx from the atmosphere, sustaining low $\hbox {pCO}_{2}$ in surface waters. This process led to modeled $\hbox {pCO}_{2}$ undersaturation, still evident at DSR = 45, particularly under low wind speed conditions (Fig. 7d). Similar to Petermann Fjord, freshwater inputs were assumed to be lower during the post-sea-ice melt stage ($\hbox {DSR} > 0$) in Sherard Osborn Fjord. However, the presence of a multi-year sea-ice edge at the fjord entrance^21^ may have restricted freshwater outflow, thereby enhancing upper-ocean stratification in the system. Glacial meltwater reduced TA in the upper-water, thereby weakening the buffering capacity of the system^75^. As a result, the fjord waters became increasingly sensitive to $\hbox {pCO}_{2}$ changes, with reduced buffering capacity leading to greater surface water acidification as $\hbox {CO}_{2}$ uptake intensified^8^ in this particular case (i.e., summer 2019 for Sherard Osborn Fjord^21^). During the following stages of $\hbox {DSR} > 0$, the absence of significant $\hbox {CO}_{2}$ uptake by primary production combined with rising temperatures and ongoing air-sea $\hbox {CO}_{2}$ exchange, resulted in sustained rise in sea surface modeled $\hbox {pCO}_{2}$ (Fig. 7c).

## Discussion

While we provide a limited snapshot of the northwestern Greenland region’s oceanographic conditions and $\hbox {pCO}_{2}^{sw}$ during a few weeks in the summer of 2019, our study represents a comprehensive examination of carbon dynamics in this remote area. We used the TML of each water column profile during the summer season as a proxy for the preceding winter surface water conditions. This, combined with the properties of summer surface water, allows us to explore the seasonal changes of $\hbox {pCO}_{2}^{sw}$ from the onset of sea-ice melt to the sampling period and the processes responsible for these changes. The two fjords surveyed in summer 2019, hosting the marine-terminating Petermann Glacier and Ryder Glacier, have previously been explored in terms of their complex and varying circulation and surface water patterns^20,21^, which showed that the summer of 2019 was marked by an anomalously large ice melt and surface water temperatures reaching $4\,^{\circ }\hbox {C}$ in Sherard Osborn Fjord. Extensive freshwater inputs in combination with the presence of multiyear sea-ice at the entrance of the fjord led to a strongly stratified surface layer with salinity values as low as 15 g $\hbox {kg}^{-1}$,^21^ in the system. In contrast, the survey underway in Petermann Fjord showed different oceanographic conditions characterized by temperature below $0\,^{\circ }\hbox {C}$ and salinity around 30 g $\hbox {kg}^{-1}$. Associated with this large spatial variability in surface water physical properties, $\hbox {pCO}_{2}^{sw}$ was also expected to show a heterogeneous distribution due to variations in freshwater inputs, temperature, biological activity, gas exchange, and physical ocean circulation processes^22,30,73,76–82^. Consequently, our analysis of the drivers of seasonal $\hbox {pCO}_{2}^{sw}$ changes from the onset of sea-ice melt to late summer reveals significant spatial differences among the different components, i.e. thermal ($\Delta pCO_{2}^{T}$), biological ($\Delta pCO_{2}^{B}$), freshwater ($\Delta pCO_{2}^{FW}$), and residuals ($\Delta pCO_{2}^{R}$), the latter including air-sea $\hbox {CO}_{2}$ fluxes and physical ocean circulation processes.

### How do physical and biogeochemical processes impact $\hbox {pCO}_2$ from sea-ice melt onset to late summer?

Although temperature is known to influence the solubility of gases, which further alters sea surface $\hbox {pCO}_{2}$^83^, no obvious relationship between $\hbox {pCO}_{2}^{sw}$ and temperature was found in surface waters at the sampling time, in individual areas, except in the Nares Strait. However, quantitatively and as expected, $\Delta pCO_{2}^{T}$ was more pronounced in Sherard Osborn Fjord compared to the rest of the region, reflecting the unique surface ocean conditions experienced by the fjord during summer 2019^21^. This high magnitude of $\Delta pCO_{2}^{T}$ is challenging to compare with other regions, as to our knowledge, no such conditions have been reported in a Greenlandic fjord. In the three other areas, $\Delta pCO_{2}^{T}$ plays a relatively minor role, since temperature is not necessarily a critical factor, as reported in the Arctic Ocean^84^. These results align with those of ref.^30^, who reported a low thermal influence to the observed $\hbox {pCO}_{2}^{sw}$ concentrations in summer. One reason for the significant temperature influence in Sherard Osborn Fjord reflect the very low sea-ice cover during the sampling time^21^, facilitating air-sea heat flux into the ocean^85,86^.

As the ice retreats, freshwater released from a combination of glacial and sea-ice melt enhances the stability of the water column and leads to a significant dilution of surface water $\hbox {pCO}_{2}$^29,87^. Freshwater inputs had only a small relative effect on $\hbox {pCO}_{2}^{sw}$ in the Petermann Fjord and the Nares Strait, which reflects the rather modest seasonal difference in salinity between the surface waters of winter and summer. In contrast, the dilution effect of freshwater inputs significantly affected surface waters in Sherard Osborn Fjord, where $\Delta pCO_{2}^{FW}$ represents the main contributor to the $\hbox {pCO}_{2}^{sw}$ changes from the onset of the sea-ice melting to late summer.

In addition, biological activity was a significant driver of $\hbox {pCO}_{2}$ drawdown in the whole region, despite surface *chl-a* values being generally low, with the highest values recorded in surface waters of the Nares Strait and Lincoln Sea (Fig. 2c). The cruise took place after substantial ice melt, when biological activity has likely migrated deeper to 20–40 m depth^88,89^, beyond the reach of underway surface sampling at 7 m. Vertical fluorescence data confirm this, showing peak values of 10 mg $\hbox {m}^{-2}$ at 30 m depth in the Lincoln Sea, and at 20 m depth in the Nares Strait and Petermann Fjord (Supplementary Figure 6). While surface *chl-a* levels did not significantly correlate with $\hbox {pCO}_{2}^{sw}$, high $\Delta pCO_{2}^{B}$ in the Lincoln Sea and the Nares Strait is likely indicative of residual impacts from early-season primary production and subsequent $\hbox {CO}_{2}$ drawdown. The estimated NCP values were 1.3 ± 0.8 mol C $\hbox {m}^{-2}$, 0.6 ± 0.4 mol C $\hbox {m}^{-2}$, 1.6 ± 1 mol C $\hbox {m}^{-2}$ and 2.9 ± 2 mol C $\hbox {m}^{-2}$ in Sherard Osborn Fjord, Peterman Fjord, the Nares Strait and the Lincoln Sea, respectively. The highest NCP was recorded in the Lincoln Sea, followed by the Nares Strait, consistent with intense spring blooms previously documented in the region north of Ellesmere Island^90–92^. In contrast, both fjords exhibited lower NCP ($<1.5$ mol C $\hbox {m}^{-2}$), with the lowest estimate in Petermann Fjord, aligning with findings from other Arctic fjords^93^. Despite low NCP values in Sherard Osborn and Petermann Fjords, $\Delta pCO_{2}^{B}$ estimates indicate that episodic biological activity might have occurred earlier in the season, especially given the longer landfast ice coverage in these systems compared to offshore regions with mobile sea-ice^94^. Interestingly, the lower NCP in Petermann Fjord compared to Sherard Osborn Fjord raises questions as to why. One possibility is that early-season sea-ice melt diluted $\hbox {pCO}_{2}^{sw}$, limiting $\hbox {CO}_{2}$ availability for phytoplankton in Petermann, despite nutrient abundance, as previously reported in Arctic spring blooms^69^. Alternatively, the sea-ice conditions in the Nares Strait, which influence the sea-ice dynamics in Petermann Fjord, may have affected the position of the ice edge at the fjord entrance earlier in the season, thereby influencing local primary productivity^94^. In 2019, early and extensive ice melt, exacerbated by an anomalously warm summer^21^, may have accelerated snow-to-melt-pond transitions, reducing ice albedo and increasing light penetration, a key trigger for under-ice blooms^95–98^. Although these bottom ice algae have been shown to exert minimal control on $\hbox {pCO}_{2}^{sw}$^99^, short-lived under-ice phytoplankton blooms may have impacted $\hbox {pCO}_{2}^{sw}$ before ice melt in Petermann and Sherard Osborn fjords. Variability in primary production across fjords is shaped by differences in surface ocean stratification and ocean-shelf interactions^100,101^. Consequently, broad generalizations about Arctic fjord productivity remain challenging due to limited data and regional differences in seasonal timing^102^. Field observations have shown that glacier fjords exhibit a wide range of productivity, from very low ($<40$ mg C $\hbox {m}^{-2}$ $\hbox {d}^{-1}$) to moderately productive ($>500$ mg C $\hbox {m}^{-2}$ $\hbox {d}^{-1}$) during the melt season^103–105^. Therefore, the episodic nature of phytoplankton blooms and their spatial heterogeneity likely explain discrepancies in primary production between the two neighboring fjords in our study. However, in Petermann Fjord, the relatively low $\Delta pCO_{2}^{T}$ and $\Delta pCO_{2}^{FW}$ compared to $\Delta pCO_{2}^{B}$ aligns with patterns observed in other Arctic fjords^30,106^, even if the 2019 summer observations show that the region falls within the low-productivity fjord category^93^.

During the sampling period, estimated $\hbox {CO}_2$ uptake rates were approximately –12 mmol $\hbox {m}^{-2}$ $\hbox {d}^{-1}$ in the Nares Strait and Petermann Fjord, –6 mmol $\hbox {m}^{-2}$ $\hbox {d}^{-1}$ in the Lincoln Sea, and –7 mmol $\hbox {m}^{-2}$ $\hbox {d}^{-1}$ in Sherard Osborn Fjord. These values are consistent with previous estimates from northeast and southwest Greenland^27,28,30,106^, the Arctic Ocean^58,107–109^, Svalbard fjords^29,31,56^, the Canadian Arctic Archipelago^22,77^, and Baffin Bay^76^, highlighting the role of northwest Greenland as a summer $\hbox {CO}_2$ sink. Quantifying the impact of air-sea $\hbox {CO}_2$ fluxes from ice melt onset to late summer remains difficult due to a lack of seasonal data. However, comparing TML observations (representing winter surface waters) with summer surface samples show $\hbox {pCO}_2$ variability of $\sim$30 $\mu$atm–less than the variation driven by biological, thermal, and freshwater processes. This indicates that while air-sea $\hbox {CO}_2$ exchange affects $\Delta pCO_2^R$, its role might be secondary to other drivers, although it still contributes to increased $\hbox {pCO}_2^{sw}$^37^. Assuming a 25-day ice-melt period and mean summer mixed-layer depths of $\sim$12 m in Petermann Fjord, the Lincoln Sea, and the Nares Strait, and $\sim$6 m in Sherard Osborn Fjord, air-sea $\hbox {CO}_2$ uptake could raise surface DIC by $\sim$25 $\mu$mol $\hbox {kg}^{-1}$ in Petermann, Sherard Osborn, and the Nares Strait, and $\sim$10 $\mu$mol $\hbox {kg}^{-1}$ in the Lincoln Sea. This corresponds to $\hbox {pCO}_2^{sw}$ increases of $\sim$55 $\mu$atm in the Nares Strait and Petermann, $\sim$80 $\mu$atm in Sherard Osborn, and $\sim$30 $\mu$atm in the Lincoln Sea. These changes likely contribute to the estimate $\Delta pCO_2^R$, although uncertainties remain.

In the Nares Strait and the Lincoln Sea, advection of sea-ice from the Arctic Ocean causes alternating ice-covered and open-water conditions that influence $\hbox {CO}_2$ exchange^110^. Under-ice processes, such as brine rejection, can increase $\hbox {pCO}_2^{sw}$, especially when not compensated by meltwater dilution or photosynthesis^89^. These processes may occur locally or result from water mass advection from north of the Nares Strait ice arch, where perennial sea-ice persists. Unaccounted physical factors such as vertical mixing and lateral advection likely also influence $\hbox {pCO}_2^{sw}$ variability^111^. In particular, the Lincoln Sea may be affected by southward advection of $\hbox {CO}_2$-rich Pacific-origin waters, reflected in high positive $\Delta pCO_2^R$ values. Conversely, Petermann Fjord exhibited a mean negative residual (Fig. 6), suggesting net $\hbox {CO}_{2}$ removal from surface waters. While calculation uncertainties certainly contribute, hydrographic connectivity between the Nares Strait and Petermann Fjord might have contributed to the estimated $\Delta pCO_{2}^{R}$. Under mobile sea-ice conditions during summer, eddy-driven exchange at the entrance of Petermann Fjord may enhance ventilation, consistent with previous findings on circulation patterns in the region^112,113^. Thus the Nares Strait as a major conduit for Arctic freshwater^114^, likely advected low-$\hbox {pCO}_{2}$ surface waters into Petermann Fjord, influencing the estimated residuals. This mechanism might also be relevant for Sherard Osborn Fjord, however, the presence of a multiyear sea-ice edge at its entrance, may have restricted advection, potentially explaining the positive residuals in Sherard Osborn Fjord but not in Petermann Fjord.

### How $\hbox {pCO}_{2}$ dynamics differ between adjacent but distinctly different fjords?

The impact of freshwater inputs on $\hbox {pCO}_{2}^{sw}$ is evident throughout the region, particularly pronounced in Sherard Osborn Fjord (Fig. 6) and, as expected, less significant and attributed to the absence of accumulation in the upper waters in Petermann Fjord^21^. The presence of strong stratification can create an optimal environment for the accumulation of $\hbox {pCO}_{2}^{sw}$ in the upper ocean, which can increase the role of freshwater inputs in carbon dynamics, and eventually reduce the buffering capacity of surface waters^8,36,115^. Conversely to Sherard Osborn Fjord, the ocean conditions of Petermann Fjord were similar to most Greenlandic fjords, highlighting the increase in biological $\hbox {CO}_{2}$ uptake as a key control of $\hbox {pCO}_{2}^{sw}$ seasonality, consistent with the studies of ref.^30^, but slightly different from that of ref.^27^, where the effect of freshwater inputs was identified as the dominant driver. Although the freshwater term being the main first-order control on $\hbox {pCO}_{2}^{sw}$ in Sherard Osborn Fjord, the overall robust seasonal $\hbox {pCO}_{2}^{sw}$ decrease is also mainly associated with a large $\hbox {CO}_{2}$ uptake from biology. This is interesting as no signal of primary productivity was observed during the time of sampling–the highly stratified, warm, and calcium carbonate-corrosive surface waters of the fjord^21^ could have ended the summer bloom of phytoplankton earlier than normal or limited the formation of a significant bloom^116^ as explained in the previous section.

In the ice-free Sherard Osborn Fjord, a localized area displayed relatively high $\hbox {pCO}_{2}^{sw}$ values ranging from 300 to 380 $\mu$atm, strongly contrasting with values below 280 $\mu$atm in the rest of the fjord. These observations of elevated $\hbox {pCO}_{2}^{sw}$ values at its inner part likely reveal a dynamic response of $\hbox {pCO}_{2}^{sw}$ to significant ice melt. Surface ocean warming can partially explain the increase in $\hbox {pCO}_{2}^{sw}$, yet when normalized to the mean fjord temperature (*n*$\hbox {pCO}_{2}^{sw}$), the values remained significantly high locally (Supplementary Figure 7), highlighting the influence of other processes. Based on calculations of each driver’s contribution in Sherard Osborn Fjord, the subsequent mixing of seawater with low $\hbox {pCO}_{2}$ freshwater and biological $\hbox {CO}_{2}$ uptake would decrease $\hbox {pCO}_{2}^{sw}$ by -120 ± 40 $\mu$atm and -74 ± 46 $\mu$atm, respectively (Fig. 6). The surface temperature increases from -1.5 to $4\,^{\circ }\hbox {C}$ from the onset of sea-ice melting to summer would raise $\hbox {pCO}_{2}^{sw}$ by approximately 62 ± 23 $\mu$atm (Fig. 6), balancing the reduction due to freshwater inputs and net biological productivity by only $\sim$30 %.

In Sherard Osborn Fjord, the other main factors that contribute to the increase in $\hbox {pCO}_{2}^{sw}$ are the processes governing the estimated residual effects ($\Delta pCO_{2}^{R}$), including atmospheric $\hbox {CO}_{2}$ fluxes and $\hbox {CO}_{2}$ replenishment driven by oceanic circulation. Among the potential physical mechanisms associated with oceanic circulation, vertical mixing has been shown to significantly influence $\hbox {pCO}_{2}^{sw}$ by transporting subsurface waters rich in dissolved inorganic carbon to the surface^25,77,79,84^. However, in the absence of detailed hydrographic time series, the presence of such processes cannot be directly assessed. Furthermore, previous studies have suggested that upwelling is typically highly localized and exerts only minor influences on larger-scale $\hbox {pCO}_{2}^{sw}$,^117^. Thus, we assume that the main factor, among the estimated residual effects, driving the increase in $\hbox {pCO}_{2}^{sw}$ is atmospheric $\hbox {CO}_{2}$ uptake during the ice-free period. When recalculated using the CO2SYS program, $\hbox {pCO}_{2}^{sw}$ is expected to rise by approximately 360 $\mu$atm at a surface water temperature of $4\,^{\circ }\hbox {C}$, aligning with the high $\hbox {pCO}_{2}^{sw}$ levels observed in the inner part of the fjord (Fig. 2d). This atmospheric $\hbox {CO}_{2}$ control mechanism is facilitated by the shallow mixed-layer depth ($\sim$6 m) and strong stratification, allowing for rapid equilibration with the atmosphere (Fig. 7).

Northern Greenland represents an area with a heavy perennial sea-ice cover, projected to be the last perennial ice refuge in the Arctic Ocean^118^. However, as the Arctic Ocean warms and the summer sea-ice disappears, strong seasonal stratification in newly ice-free regions could become a dominant feature over time. Furthermore, the observed thinning of sea-ice^119^ can lead to a decrease in sea-ice export^120^, which means it is unclear whether areas dominated by ice export will experience an increase or a decrease in surface ocean stratification. As sea surface temperatures continue to increase after the complete melting of sea-ice during summer, the surface ocean $\hbox {CO}_{2}$ uptake capacity would further decrease due to the warming effect on surface water $\hbox {pCO}_{2}^{sw}$. Here, we demonstrate that the increase in $\hbox {CO}_{2}$ drawdown from freshwater forcing surpasses the reduced uptake caused by the warming of the mixed-layer in Sherard Osborn Fjord. If Sherard Osborn Fjord, with its shallow, fresh and warm surface mixed-layer, is considered an analog of future coastal conditions in a warming Arctic Ocean, we could expect these areas to efficiently absorb atmospheric $\hbox {CO}_{2}$ in the future. However, this $\hbox {CO}_{2}$ uptake would quickly diminish due to rapid equilibration with the atmosphere, driven by the strongly stratified mixed-layer, surface warming and low biological $\hbox {CO}_{2}$ fixation. In summary, our study provides valuable information on the seasonality of $\hbox {pCO}_{2}^{sw}$ in four distinct coastal regions of northern Greenland. Analysis of influencing factors, including thermal effects, biological activity, freshwater inputs and air-sea $\hbox {CO}_{2}$ fluxes, reveals the complex interplay of these processes in driving the observed patterns of surface seawater $\hbox {pCO}_{2}^{sw}$. Our findings contribute to the growing body of knowledge on the carbonate system dynamics of Greenland’s coastal regions, emphasizing the need for further research to better understand and quantify the impacts of physical and biogeochemical processes on the marine carbon cycle in the rapidly changing Arctic environment.

## Electronic supplementary material

Supplementary Material 1

## Data Availability

The data presented in this paper are available from the Bolin Centre for Climate Research and the USGS ScienceBase databases. These include: - CTD sensor data: https://bolin.su.se/data/ryder-2019-ctd - Underway measurements of seawater pH and total alkalinity: https://bolin.su.se/data/ryder-2019-surface-seawater-ph-alkalinity - Seawater carbonate chemistry from CTD bottle samples: https://bolin.su.se/data/ryder-2019-ctd-bottle-ph-alkalinity - Underway measurements of chlorophyll a: https://www.sciencebase.gov/catalog/item/669955b7d34e9ac16e164c82

## References

[1] Rantanen, M. et al. The arctic has warmed nearly four times faster than the globe since 1979. *Commun. Rarth Environ.* **3**, 168 (2022).

[2] Kwok, R. et al. Thinning and volume loss of the arctic ocean sea ice cover: 2003–2008. *J. Geophys. Res. Oceans* **114**, 145 (2009).

[3] Stroeve, J. C. et al. The arctic’s rapidly shrinking sea ice cover: A research synthesis. *Clim. Change* **110**, 1005–1027 (2012).

[4] Onarheim, I. H., Eldevik, T., Smedsrud, L. H. & Stroeve, J. C. Seasonal and regional manifestation of arctic sea ice loss. *J. Climate* **31**, 4917–4932 (2018).

[5] Rignot, E., Velicogna, I., van den Broeke, M. R., Monaghan, A. & Lenaerts, J. T. Acceleration of the contribution of the Greenland and Antarctic ice sheets to sea level rise. *Geophys. Res. Lett.* **38**, 1424 (2011).

[6] Mouginot, J. et al. Forty-six years of greenland ice sheet mass balance from 1972 to 2018. *Proc. Natl. Acad. Sci.* **116**, 9239–9244 (2019). 31010924 10.1073/pnas.1904242116 PMC6511040

[7] Déry, S. J., Stadnyk, T. A., MacDonald, M. K. & Gauli-Sharma, B. Recent trends and variability in river discharge across northern canada. *Hydrol. Earth Syst. Sci.* **20**, 4801–4818 (2016).

[8] Cai, W.-J. et al. Decrease in the co2 uptake capacity in an ice-free arctic ocean basin. *Science* **329**, 556–559 (2010). 20651119 10.1126/science.1189338

[9] Parmentier, F.-J.W. et al. The impact of lower sea-ice extent on arctic greenhouse-gas exchange. *Nat. Clim. Chang.* **3**, 195–202 (2013).

[10] DeGrandpre, M. et al. Changes in the arctic ocean carbon cycle with diminishing ice cover. *Geophys. Res. Lett.* **47**, e2020GL088051 (2020). 32728302 10.1029/2020GL088051 PMC7380310

[11] Ouyang, Z. et al. The changing co2 sink in the western arctic ocean from 1994 to 2019. *Global Biogeochem. Cycles* **36**, e2021GB007032 (2022).

[12] Qi, D. et al. Climate change drives rapid decadal acidification in the arctic ocean from 1994 to 2020. *Science* **377**, 1544–1550 (2022). 36173841 10.1126/science.abo0383

[13] Bates, N. & Mathis, J. The arctic ocean marine carbon cycle: evaluation of air-sea co 2 exchanges, ocean acidification impacts and potential feedbacks. *Biogeosciences* **6**, 2433–2459 (2009).

[14] Yasunaka, S. et al. Mapping of the air-sea co2 flux in the arctic ocean and its adjacent seas: Basin-wide distribution and seasonal to interannual variability. *Polar Sci.* **10**, 323–334 (2016).

[15] Yasunaka, S. et al. Arctic ocean co 2 uptake: An improved multiyear estimate of the air-sea co 2 flux incorporating chlorophyll a concentrations. *Biogeosciences* **15**, 1643–1661 (2018).

[16] Yasunaka, S. et al. An assessment of co2 uptake in the arctic ocean from 1985 to 2018. *Global Biogeochem. Cycles* **37**, e2023GB007806 (2023).

[17] Rignot, E., Gogineni, S., Joughin, I. & Krabill, W. Contribution to the glaciology of northern Greenland from satellite radar interferometry. *J. Geophys. Res. Atmosph.* **106**, 34007–34019 (2001).

[18] Hill, E. A., Carr, J. R. & Stokes, C. R. A review of recent changes in major marine-terminating outlet glaciers in northern greenland. *Front. Earth Sci.* **4**, 111 (2017).

[19] Moore, G., Schweiger, A., Zhang, J. & Steele, M. Spatiotemporal variability of sea ice in the arctic’s last ice area. *Geophys. Res. Lett.* **46**, 11237–11243 (2019).

[20] Jakobsson, M. et al. Ryder glacier in northwest Greenland is shielded from warm atlantic water by a bathymetric sill. *Commun. Earth Environ.* **1**, 45 (2020).

[21] Stranne, C. et al. The climate sensitivity of northern greenland fjords is amplified through sea-ice damming. *Commun. Earth Environ.* **2**, 70 (2021).

[22] Burgers, T. M. et al. Surface water pco2 variations and sea-air co2 fluxes during summer in the eastern Canadian arctic. *J. Geophys. Res. Oceans* **122**, 9663–9678 (2017).

[23] Burgers, T. et al. Distinguishing physical and biological controls on the carbon dynamics in a high-arctic outlet strait. *J. Geophys. Res. Oceans* **128**, e2022JC019393 (2023).

[24] Azetsu-Scott, K. et al. Calcium carbonate saturation states in the waters of the Canadian arctic archipelago and the Labrador sea. *J. Geophys. Res. Oceans* **115**, c11 (2010).

[25] Kanna, N. et al. Upwelling of macronutrients and dissolved inorganic carbon by a subglacial freshwater driven plume in bowdoin fjord, northwestern greenland. *J. Geophys. Res. Biogeosci.* **123**, 1666–1682 (2018).

[26] Horikawa, T., Nomura, D., Kanna, N., Fukamachi, Y. & Sugiyama, S. Effects of the glacial meltwater supply on carbonate chemistry in Bowdoin fjord, northwestern Greenland. *Front. Mar. Sci.* **9**, 873860 (2022).

[27] Sejr, M. K. et al. Air-sea flux of co2 in arctic coastal waters influenced by glacial melt water and sea ice. *Tellus B Chem. Phys. Meteorol.* **63**, 815–822 (2011).

[28] Rysgaard, S. et al. High air-sea co2 uptake rates in nearshore and shelf areas of southern Greenland: Temporal and spatial variability. *Mar. Chem.* **128**, 26–33 (2012).

[29] Evans, W., Mathis, J. & Cross, J. Calcium carbonate corrosivity in an alaskan inland sea. *Biogeosciences* **11**, 365–379 (2014).

[30] Meire, L. et al. Glacial meltwater and primary production are drivers of strong co 2 uptake in fjord and coastal waters adjacent to the greenland ice sheet. *Biogeosciences* **12**, 2347–2363 (2015).

[31] Ericson, Y., Falck, E., Chierici, M., Fransson, A. & Kristiansen, S. Marine co2 system variability in a high arctic tidewater-glacier fjord system, Tempelfjorden, Svalbard. *Cont. Shelf Res.* **181**, 1–13 (2019).

[32] Frey, K., Comiso, J., Cooper, L., Grebmeier, J. & Stock, L. V. Arctic ocean primary productivity: The response of marine algae to climate warming and sea ice decline. *Arctic Report Card* (2021).

[33] Henson, H. C. et al. Coastal freshening drives acidification state in Greenland fjords. *Sci. Total Environ.* **855**, 158962 (2023). 36170921 10.1016/j.scitotenv.2022.158962

[34] Henson, H. C. et al. Resolving heterogeneity in co2 uptake potential in the greenland coastal ocean. *J. Geophys. Res. Biogeosci.* **129**, e2024JG008246 (2024). 39610668 10.1029/2024JG008246 PMC11600391

[35] Juul-Pedersen, T. et al. Seasonal and interannual phytoplankton production in a sub-arctic tidewater outlet glacier fjord, SW Greenland. *Mar. Ecol. Prog. Ser.* **524**, 27–38 (2015).

[36] Else, B. G. et al. Further observations of a decreasing atmospheric co2 uptake capacity in the Canada basin (arctic ocean) due to sea ice loss. *Geophys. Res. Lett.* **40**, 1132–1137 (2013).

[37] Yang, W. et al. The impact of sea ice melt on the evolution of surface p co2 in a polar ocean basin. *Front. Mar. Sci.* **11**, 1307295 (2024).

[38] Mueller, J. D. & Rehder, G. Metrology of ph measurements in brackish waters-part 2: experimental characterization of purified meta-cresol purple for spectrophotometric pht measurements. *Front. Mar. Sci.* **5**, 177 (2018).

[39] Seelmann, K., Aßmann, S. & Körtzinger, A. Characterization of a novel autonomous analyzer for seawater total alkalinity: Results from laboratory and field tests. *Limnol. Oceanogr. Methods* **17**, 515–532 (2019).

[40] Van Heuven, S., Pierrot, D., Rae, J., Lewis, E. & Wallace, D. Matlab program developed for co2 system calculations, ornl/cdiac-105b, carbon dioxide information analysis center, oak ridge national laboratory, us department of energy, oak ridge, tennessee, 10.3334/cdiac/otg.CO2SYS MATLAB v1 **1** (2011).

[41] Mehrbach, C., Culberson, C., Hawley, J. & Pytkowicx, R. Measurement of the apparent dissociation constants of carbonic acid in seawater at atmospheric pressure 1. *Limnol. Oceanogr.* **18**, 897–907 (1973).

[42] Dickson, A. & Millero, F. J. A comparison of the equilibrium constants for the dissociation of carbonic acid in seawater media. Deep Sea Research Part A. *Oceanographic Res. Papers* **34**, 1733–1743 (1987).

[43] Dickson, A. G. Thermodynamics of the dissociation of boric acid in synthetic seawater from 273.15 to 318.15 k. Deep Sea Research Part A. *Oceanogr. Res. Papers* **37**, 755–766 (1990).

[44] Orr, J. C., Epitalon, J.-M., Dickson, A. G. & Gattuso, J.-P. Routine uncertainty propagation for the marine carbon dioxide system. *Mar. Chem.* **207**, 84–107 (2018).

[45] Carter, B., Radich, J., Doyle, H. & Dickson, A. An automated system for spectrophotometric seawater ph measurements. *Limnol. Oceanogr. Methods* **11**, 16–27 (2013).

[46] Clayton, T. D. & Byrne, R. H. Spectrophotometric seawater ph measurements: total hydrogen ion concentration scale calibration of m-cresol purple and at-sea results. *Deep Sea Res. Part I* **40**, 2115–2129 (1993).

[47] Fransson, A., Engelbrektsson, J. & Chierici, M. Development and optimization of a labview program for spectrophotometric ph measurements of seawater, phspec ver 2.5. *University of Gothenburg* (2013).

[48] Chierici, M., Fransson, A. & Anderson, L. Influence of m-cresol purple indicator additions on the ph of seawater samples: correction factors evaluated from a chemical speciation model. *Mar. Chem.* **65**, 281–290 (1999).

[49] Haraldsson, C., Anderson, L. G., Hassellöv, M., Hulth, S. & Olsson, K. Rapid, high-precision potentiometric titration of alkalinity in ocean and sediment pore waters. *Deep Sea Res. Part I* **44**, 2031–2044 (1997).

[50] Tomczak, M. & Liefrink, S. Interannual variations of water mass volumes in the southern ocean. *J. Atmosph. Ocean Sci.* **10**, 31–42 (2005).

[51] Nomura, D. et al. Winter-to-summer evolution of pco 2 in surface water and air-sea co 2 flux in the seasonal ice zone of the southern ocean. *Biogeosciences* **11**, 5749–5761 (2014).

[52] Mackay, N. & Watson, A. Winter air-sea co2 fluxes constructed from summer observations of the polar southern ocean suggest weak outgassing. *J. Geophys. Res. Oceans* **126**, e2020JC016600 (2021).

[53] Millero, F. J., Lee, K. & Roche, M. Distribution of alkalinity in the surface waters of the major oceans. *Mar. Chem.* **60**, 111–130 (1998).

[54] Rysgaard, S., Bendtsen, J., Pedersen, L. T., Ramløv, H. & Glud, R. N. Increased co2 uptake due to sea ice growth and decay in the nordic seas. *J. Geophys. Res. Oceans* **114**, c9 (2009).

[55] Fransson, A. et al. Influence of glacial water and carbonate minerals on wintertime sea-ice biogeochemistry and the co2 system in an arctic fjord in svalbard. *Ann. Glaciol.* **61**, 320–340 (2020).

[56] Fransson, A. et al. Effect of glacial drainage water on the co 2 system and ocean acidification state in an a rctic tidewater-glacier fjord during two contrasting years. *J. Geophys. Res. Oceans* **120**, 2413–2429 (2015).

[57] Cooper, L. et al. Flow-weighted values of runoff tracers (18o, doc, ba, alkalinity) from the six largest arctic rivers. *Geophys. Res. Lett.* **35**, 18 (2008).

[58] Fransson, A. et al. The importance of shelf processes for the modification of chemical constituents in the waters of the eurasian arctic ocean: implication for carbon fluxes. *Cont. Shelf Res.* **21**, 225–242 (2001).

[59] Yamamoto-Kawai, M., Tanaka, N. & Pivovarov, S. Freshwater and brine behaviors in the arctic ocean deduced from historical data of 18o and alkalinity (1929–2002 ad). *J. Geophys. Res. Oceans* **110**, c110 (2005).

[60] Alkire, M. B., Jacobson, A. D., Lehn, G. O., Macdonald, R. W. & Rossi, M. W. On the geochemical heterogeneity of rivers draining into the straits and channels of the canadian arctic archipelago. *J. Geophys. Res. Biogeosci.* **122**, 2527–2547 (2017).

[61] Alkire, M. B., Falkner, K. K., Boyd, T. & Macdonald, R. W. Sea ice melt and meteoric water distributions in nares strait, baffin bay, and the canadian arctic archipelago. *J. Mar. Res.* **68**, 767–798 (2010).

[62] Jackson, J. M. et al. On the waters upstream of nares strait, arctic ocean, from 1991 to 2012. *Cont. Shelf Res.* **73**, 83–96 (2014).

[63] Wanninkhof, R. Relationship between wind speed and gas exchange over the ocean revisited. *Limnol. Oceanogr. Methods* **12**, 351–362 (2014).

[64] Weiss, R. Carbon dioxide in water and seawater: the solubility of a non-ideal gas. *Mar. Chem.* **2**, 203–215 (1974).

[65] Wanninkhof, R. & Triñanes, J. The impact of changing wind speeds on gas transfer and its effect on global air-sea co2 fluxes. *Global Biogeochem. Cycles* **31**, 961–974 (2017).

[66] Jersild, A. & Landschützer, P. A spatially explicit uncertainty analysis of the air-sea co2 flux from observations. *Geophys. Res. Lett.* **51**, e2023GL106636 (2024).

[67] Prytherch, J. et al. Direct determination of the air-sea co2 gas transfer velocity in arctic sea ice regions. *Geophys. Res. Lett.* **44**, 3770–3778 (2017).

[68] Prytherch, J. & Yelland, M. Wind, convection and fetch dependence of gas transfer velocity in an arctic sea-ice lead determined from eddy covariance co2 flux measurements. *Global Biogeochem. Cycles* **35**, e2020GB006633 (2021).

[69] Takahashi, T. et al. Global sea-air co2 flux based on climatological surface ocean pco2, and seasonal biological and temperature effects. *Deep Sea Res. Part II* **49**, 1601–1622 (2002).

[70] Friis, K., Körtzinger, A. & Wallace, D. W. The salinity normalization of marine inorganic carbon chemistry data. *Geophys. Res. Lett.* **30**, 10457 (2003).

[71] De Steur, L. et al. Hydrographic changes in the lincoln sea in the arctic ocean with focus on an upper ocean freshwater anomaly between 2007 and 2010. *J. Geophys. Res. Oceans* **118**, 4699–4715 (2013).

[72] Steele, M. et al. Circulation of summer pacific halocline water in the arctic ocean. *J. Geophys. Res. Oceans* **109**, c2 (2004).

[73] Mathis, J. T. *et al.* Storm-induced upwelling of high pco2 waters onto the continental shelf of the western arctic ocean and implications for carbonate mineral saturation states. *Geophysical Research Letters* **39** (2012).

[74] Arrigo, K. R. & van Dijken, G. L. Continued increases in arctic ocean primary production. *Prog. Oceanogr.* **136**, 60–70 (2015).

[75] Reisdorph, S. C. & Mathis, J. T. The dynamic controls on carbonate mineral saturation states and ocean acidification in a glacially dominated estuary. *Estuar. Coast. Shelf Sci.* **144**, 8–18 (2014).

[76] Miller, L. A. et al. Carbon distributions and fluxes in the north water, 1998 and 1999. *Deep Sea Res. Part II* **49**, 5151–5170 (2002).

[77] Fransson, A., Chierici, M. & Nojiri, Y. New insights into the spatial variability of the surface water carbon dioxide in varying sea ice conditions in the arctic ocean. *Cont. Shelf Res.* **29**, 1317–1328 (2009).

[78] Mucci, A., Lansard, B., Miller, L. A. & Papakyriakou, T. N. Co2 fluxes across the air-sea interface in the southeastern beaufort sea: Ice-free period. *Journal of Geophysical Research: Oceans* **115** (2010).

[79] Else, B. *et al.* Annual cycles of pco2sw in the southeastern beaufort sea: New understandings of air-sea co2 exchange in arctic polynya regions. *Journal of Geophysical Research: Oceans* **117** (2012).

[80] Geilfus, N.-X. et al. Spatial and temporal variability of seawater pco2 within the canadian arctic archipelago and baffin bay during the summer and autumn 2011. *Cont. Shelf Res.* **156**, 1–10 (2018).

[81] Ahmed, M. M. et al. Widespread surface water p co2 undersaturation during ice-melt season in an arctic continental shelf sea (hudson bay, canada). *Elem Sci Anth* **9**, 00130 (2021).

[82] Roobaert, A., Resplandy, L., Laruelle, G. G., Liao, E. & Regnier, P. Unraveling the physical and biological controls of the global coastal co2 sink. *Global Biogeochem. Cycles* **38**, e2023GB007799 (2024).

[83] Takahashi, T., Olafsson, J., Goddard, J. G., Chipman, D. W. & Sutherland, S. Seasonal variation of co2 and nutrients in the high-latitude surface oceans: A comparative study. *Global Biogeochem. Cycles* **7**, 843–878 (1993).

[84] Chierici, M. et al. Impact of biogeochemical processes and environmental factors on the calcium carbonate saturation state in the circumpolar flaw lead in the amundsen gulf, arctic ocean. *Journal of Geophysical Research: Oceans* **116**, (2011).

[85] Jenkins, M. & Dai, A. The impact of sea-ice loss on arctic climate feedbacks and their role for arctic amplification. *Geophys. Res. Lett.* **48**, e2021GL094599 (2021).

[86] Rysgaard, S. et al. Sea ice contribution to the air-sea co2 exchange in the arctic and southern oceans. *Tellus B Chem. Phys. Meteorol.* **63**, 823–830 (2011).

[87] Geilfus, N.-X. et al. Inorganic carbon dynamics of melt-pond-covered first-year sea ice in the canadian arctic. *Biogeosciences* **12**, 2047–2061 (2015).

[88] Martin, J. et al. Prevalence, structure and properties of subsurface chlorophyll maxima in canadian arctic waters. *Mar. Ecol. Prog. Ser.* **412**, 69–84 (2010).

[89] Burgers, T. M., Tremblay, J.-E., Else, B. G. & Papakyriakou, T. N. Estimates of net community production from multiple approaches surrounding the spring ice-edge bloom in baffin bay. *Elementa: Science of the Anthropocene* **8** (2020).

[90] Klein, B. et al. Phytoplankton biomass, production and potential export in the north water. *Deep Sea Res. Part II* **49**, 4983–5002 (2002).

[91] Odate, T. et al. Temporal and spatial patterns in the surface-water biomass of phytoplankton in the north water. *Deep Sea Res. Part II* **49**, 4947–4958 (2002).

[92] Marchese, C. et al. Changes in phytoplankton bloom phenology over the north water (now) polynya: a response to changing environmental conditions. *Polar Biol.* **40**, 1721–1737 (2017).

[93] Simo-Matchim, A.-G., Gosselin, M., Blais, M., Gratton, Y. & Tremblay, J. -É. Seasonal variations of phytoplankton dynamics in nunatsiavut fjords (labrador, canada) and their relationships with environmental conditions. *J. Mar. Syst.* **156**, 56–75 (2016).

[94] Detlef, H. et al. Holocene sea-ice dynamics in petermann fjord. *The Cryosphere Discussions* **2021**, 1–40 (2021).

[95] Arrigo, K. R. et al. Massive phytoplankton blooms under arctic sea ice. *Science* **336**, 1408–1408 (2012). 22678359 10.1126/science.1215065

[96] Arrigo, K. R. et al. Phytoplankton blooms beneath the sea ice in the chukchi sea. *Deep Sea Res. Part II* **105**, 1–16 (2014).

[97] Oziel, L. et al. Environmental factors influencing the seasonal dynamics of spring algal blooms in and beneath sea ice in western baffin bay. *Elem Sci Anth* **7**, 34 (2019).

[98] Ardyna, M. et al. Under-ice phytoplankton blooms: Shedding light on the invisible part of arctic primary production. *Front. Mar. Sci.* **7**, 608032 (2020).

[99] Else, B. G. et al. Response of the arctic marine inorganic carbon system to ice algae and under-ice phytoplankton blooms: A case study along the fast-ice edge of baffin bay. *J. Geophys. Res. Oceans* **124**, 1277–1293 (2019).

[100] Straneo, F. & Cenedese, C. The dynamics of greenland’s glacial fjords and their role in climate. *Ann. Rev. Mar. Sci.* **7**, 89–112 (2015). 25149564 10.1146/annurev-marine-010213-135133

[101] Jackson, R. H., Lentz, S. J. & Straneo, F. The dynamics of shelf forcing in greenlandic fjords. *J. Phys. Oceanogr.* **48**, 2799–2827 (2018).

[102] Hopwood, M. J. et al. Non-linear response of summertime marine productivity to increased meltwater discharge around greenland. *Nat. Commun.* **9**, 3256 (2018). 30108210 10.1038/s41467-018-05488-8 PMC6092443

[103] Jensen, H. M., Pedersen, L., Burmeister, A. & Winding Hansen, B. Pelagic primary production during summer along 65 to 72 n off west greenland. *Polar Biol.* **21**, 269–278 (1999).

[104] Hop1, H., et al. The marine ecosystem of kongsfjorden, svalbard. *Polar Res.* **21**, 167–208 (2002).

[105] Meire, L. et al. Marine-terminating glaciers sustain high productivity in greenland fjords. *Glob. Change Biol.* **23**, 5344–5357 (2017). 10.1111/gcb.13801 28776870

[106] Sejr, M. K. et al. Seasonal dynamics of autotrophic and heterotrophic plankton metabolism and pco2 in a subarctic greenland fjord. *Limnol. Oceanogr.* **59**, 1764–1778 (2014).

[107] Bates, N. R. Air-sea co2 fluxes and the continental shelf pump of carbon in the chukchi sea adjacent to the arctic ocean. *Journal of Geophysical Research: Oceans* **111** (2006).

[108] Murata, A. & Takizawa, T. Summertime co2 sinks in shelf and slope waters of the western arctic ocean. *Cont. Shelf Res.* **23**, 753–776 (2003).

[109] Evans, W. et al. Sea-air co2 exchange in the western arctic coastal ocean. *Global Biogeochem. Cycles* **29**, 1190–1209 (2015).

[110] Rabe, B., Münchow, A., Johnson, H. & Melling, H. Nares strait hydrography and salinity field from a 3-year moored array. *Journal of Geophysical Research: Oceans* **115** (2010).

[111] Islam, F. et al. Sea surface p co 2 and o 2 dynamics in the partially ice-covered a rctic o cean. *J. Geophys. Res. Oceans* **122**, 1425–1438 (2017).

[112] Johnson, H., Münchow, A., Falkner, K. & Melling, H. Ocean circulation and properties in petermann fjord, greenland. *J. Geophys. Res. Oceans* **116** (2011).

[113] Shroyer, E. L., Padman, L., Samelson, R., Münchow, A. & Stearns, L. A. Seasonal control of petermann gletscher ice-shelf melt by the ocean’s response to sea-ice cover in nares strait. *J. Glaciol.* **63**, 324–330 (2017).

[114] Münchow, A. Volume and freshwater flux observations from nares strait to the west of greenland at daily time scales from 2003 to 2009. *J. Phys. Oceanogr.* **46**, 141–157 (2016).

[115] Bates, N. R. et al. A time-series view of changing surface ocean chemistry due to ocean uptake of anthropogenic co2 and ocean acidification. *Oceanography* **27**, 126–141 (2014).

[116] von Appen, W.-J. et al. Sea-ice derived meltwater stratification slows the biological carbon pump: results from continuous observations. *Nat. Commun.* **12**, 7309 (2021). 34911949 10.1038/s41467-021-26943-z PMC8674288

[117] Ahmed, M., Else, B., Burgers, T. & Papakyriakou, T. Variability of surface water pco2 in the canadian arctic archipelago from 2010 to 2016. *J. Geophys. Res. Oceans* **124**, 1876–1896 (2019).

[118] Wang, M. & Overland, J. E. A sea ice free summer arctic within 30 years?. *Geophys. Res. Lett.* **36**, 16 (2009).

[119] Belter, H. J. et al. Interannual variability in transpolar drift summer sea ice thickness and potential impact of atlantification. *Cryosphere* **15**, 2575–2591 (2021).

[120] Zamani, B., Krumpen, T., Smedsrud, L. H. & Gerdes, R. Fram strait sea ice export affected by thinning: comparing high-resolution simulations and observations. *Clim. Dyn.* **53**, 3257–3270 (2019).

[121] Rignot, E. & Mouginot, J. Ice flow in greenland for the international polar year 2008–2009. *Geophys. Res. Lett.* **39**, 11 (2012).

[122] Jakobsson, M. et al. The international bathymetric chart of the arctic ocean version 4.0. *Sci. Data* **7**, 176 (2020). 32647176 10.1038/s41597-020-0520-9 PMC7347603

