# Supplementary material for: Unraveling ocean pCO2 dynamics in Northwest Greenland Fjords
Source: Sci Rep. 2025 Aug 11;15:29341. doi: 10.1038/s41598-025-12720-1 (PMC12339947; doi:10.1038/s41598-025-12720-1)
Supplement: Supplementary file 1 — Supplementary Material 1 [file 41598_2025_12720_MOESM1_ESM.pdf]

Supplementary Figures :

Unraveling ocean pCO_2 dynamics in Northwest Greenland Fjords

Camille Hayatte Akhoudas[1,2*], Adam Ulfsbo[3], Brett F. Thornton[1,2], John W. Pohlman[4], Lee-Gray Boze[4], Martin Jakobsson[1,2], and Christian Stranne[1,2]

[1]Department of Geological Sciences, Stockholm University, Stockholm, Sweden

[2]Bolin Centre for Climate Research, Stockholm University, Stockholm, Sweden

[3]Department of Marine Sciences, University of Gothenburg, Gothenburg, Sweden

[4]U.S. Geological Survey, Woods Hole Coastal and Marine Science Center, Woods Hole, MA, United States

Supplementary Figures

Accompanying the article "Unraveling ocean pCO_2 dynamics in Northwest Greenland Fjords" by Akhoudas, CH., Ulfsbo A., Thornton, B. F., Pohlman J. W., Boze LG., Jakobsson M. and Stranne, C.

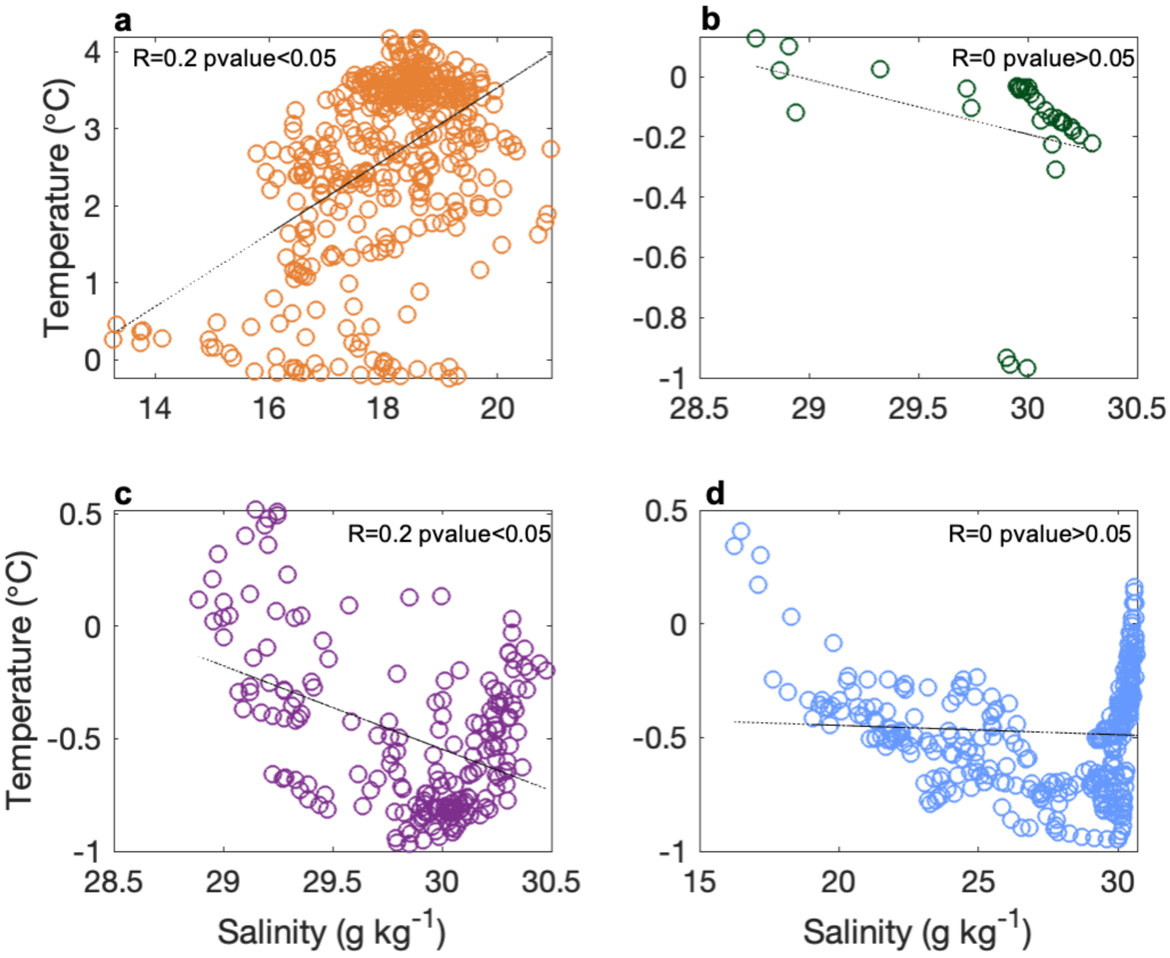

Figure 1. Relationships between surface seawater temperature and salinity in Sherard Osborn Fjord (a), Petermann Fjord (b), Nares Strait (c) and Lincoln Sea (d). Black lines indicate linear regressions, associated with their coefficient correlations between 0 and 0.2 and their p-values.

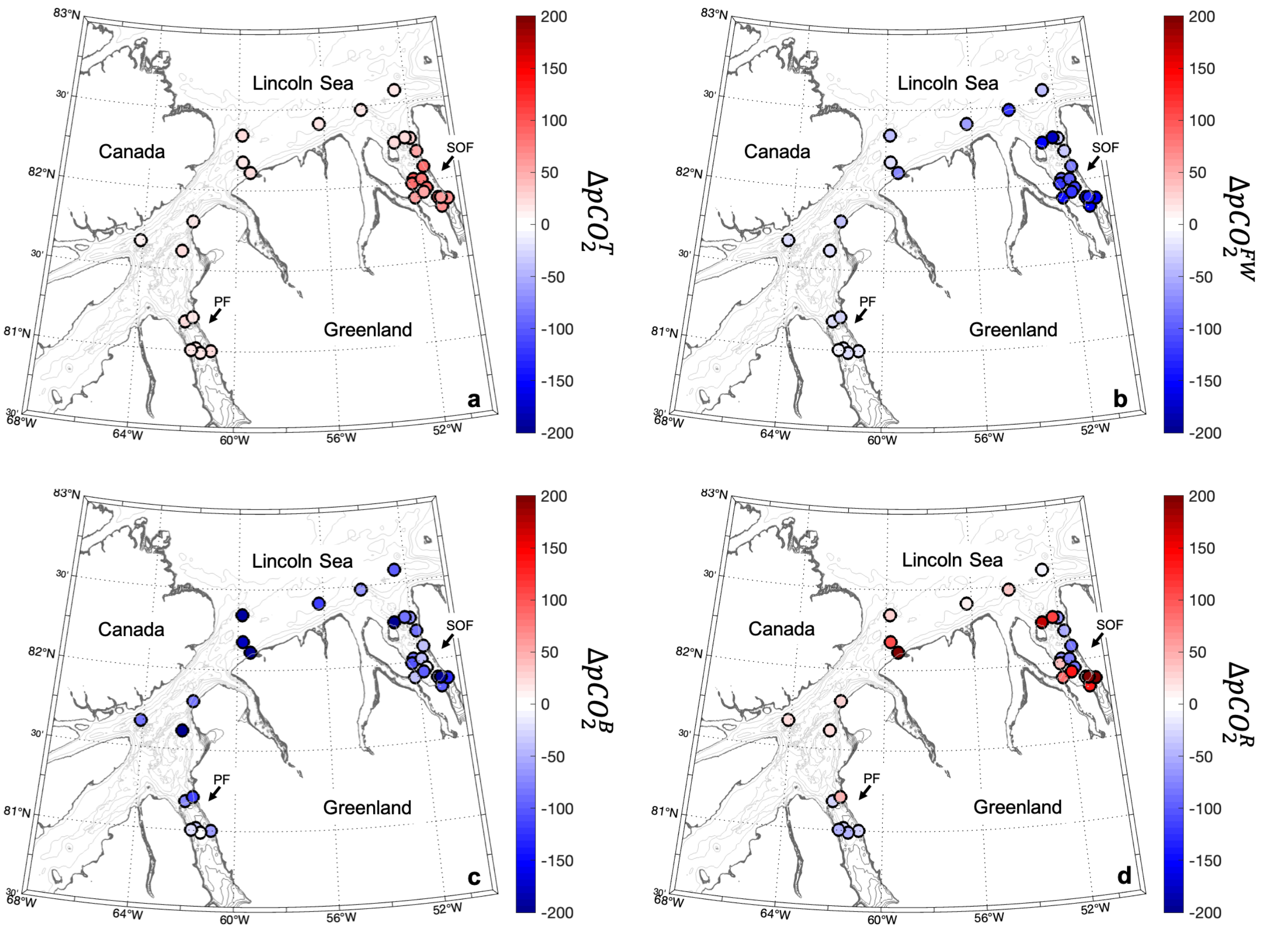

Figure 2. Spatial distribution of changes in surface seawater pCO_2^{sw} due to temperature, biological, freshwater inputs and residuals effects. PF corresponds to Petermann Fjord and SOF corresponds to Sherard Osborn Fjord.

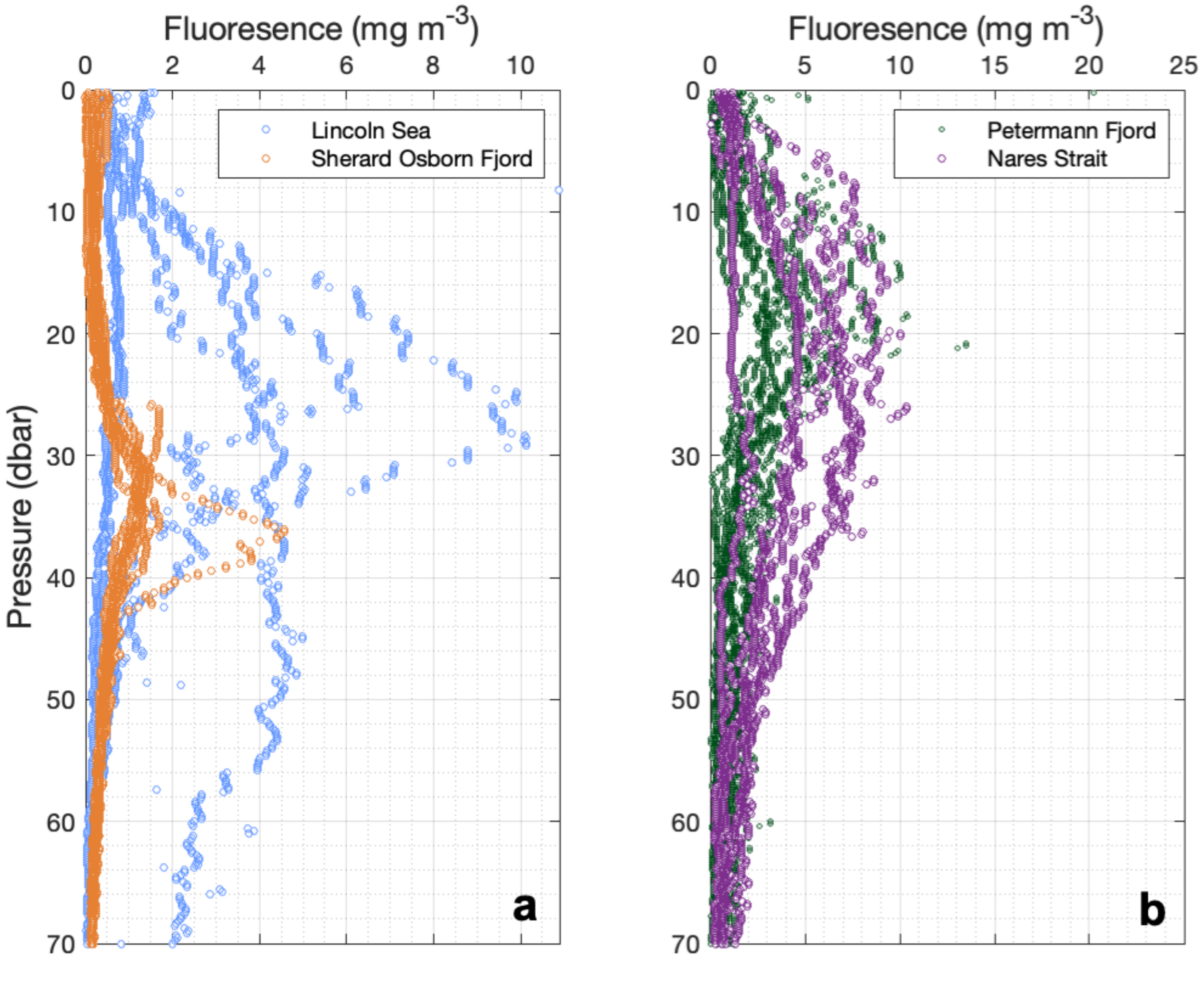

Figure 3. Fluorescence profiles Sherard Osborn Fjord (orange), Lincoln Sea (blue), Nares Strait (purple), Petermann Fjord (green).

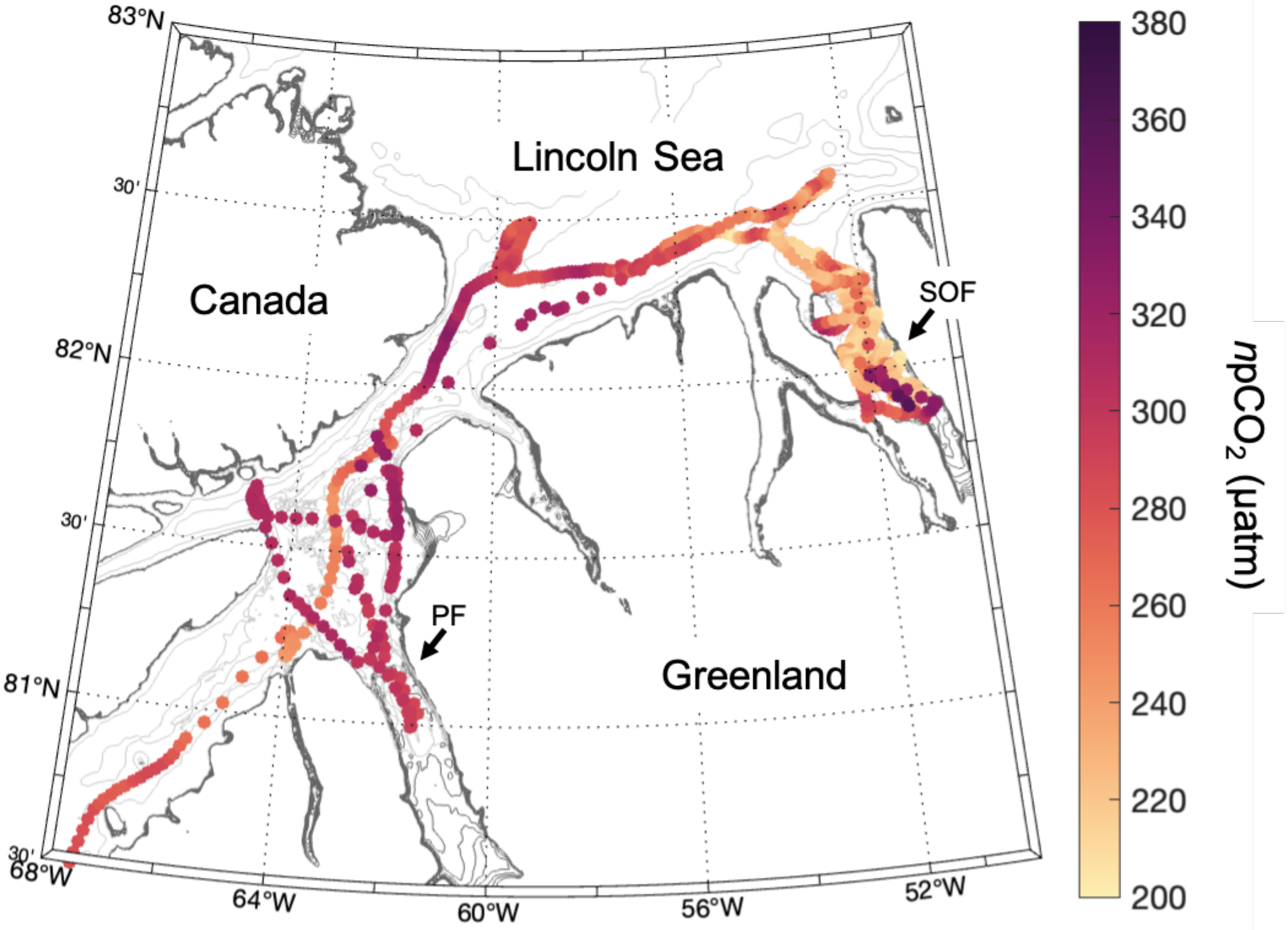

Figure 4. Spatial distribution of underway calculated pCO_2^{sw} corrected for the temperature effect. PF corresponds to Petermann Fjord and SOF corresponds to Sherard Osborn Fjord.

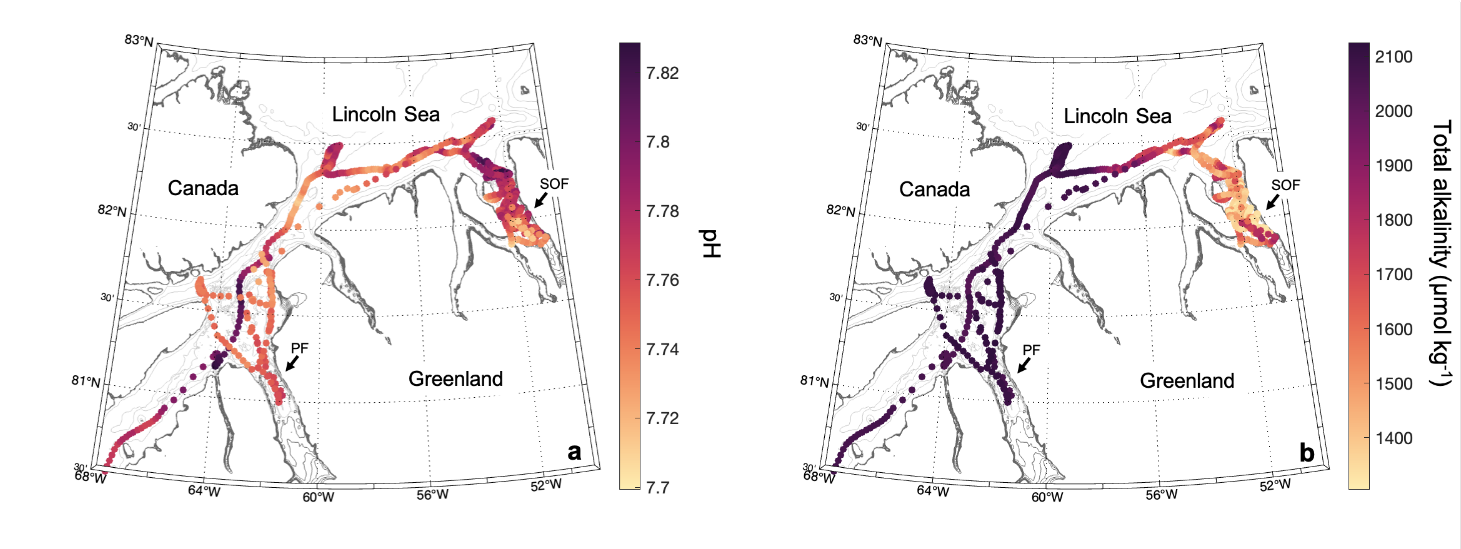

Figure 5. Spatial distribution of underway measurements of surface pH and total alkalinity. PF corresponds to Petermann Fjord and SOF corresponds to Sherard Osborn Fjord.

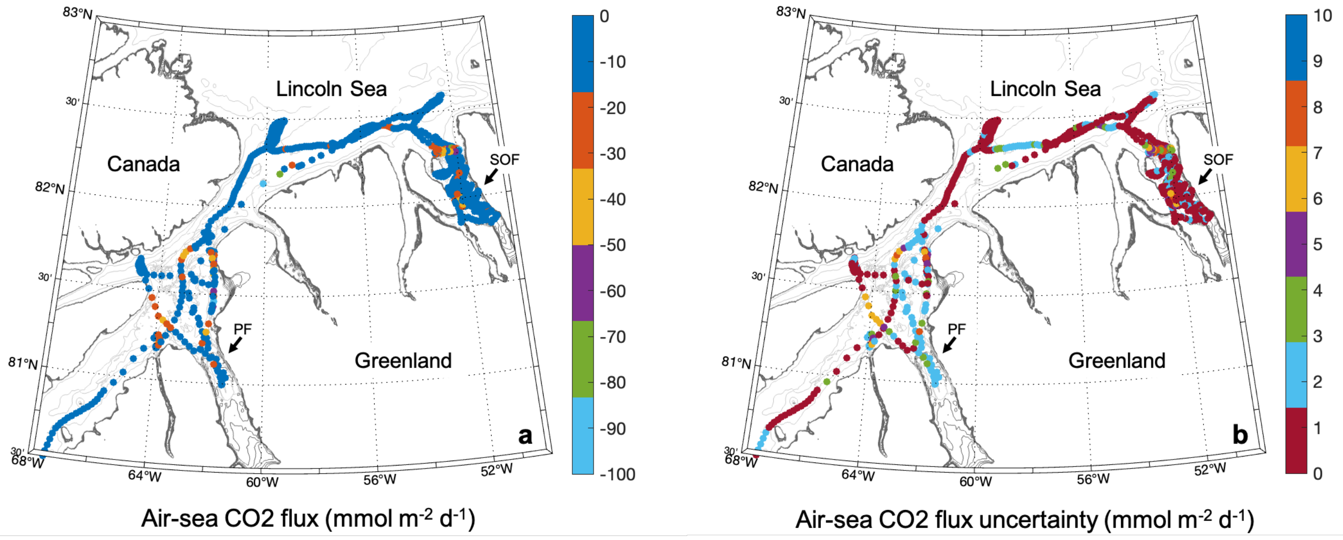

Figure 6. Spatial distribution of air-sea CO_2 flux and its associated estimated uncertainty. PF corresponds to Petermann Fjord and SOF corresponds to Sherard Osborn Fjord.

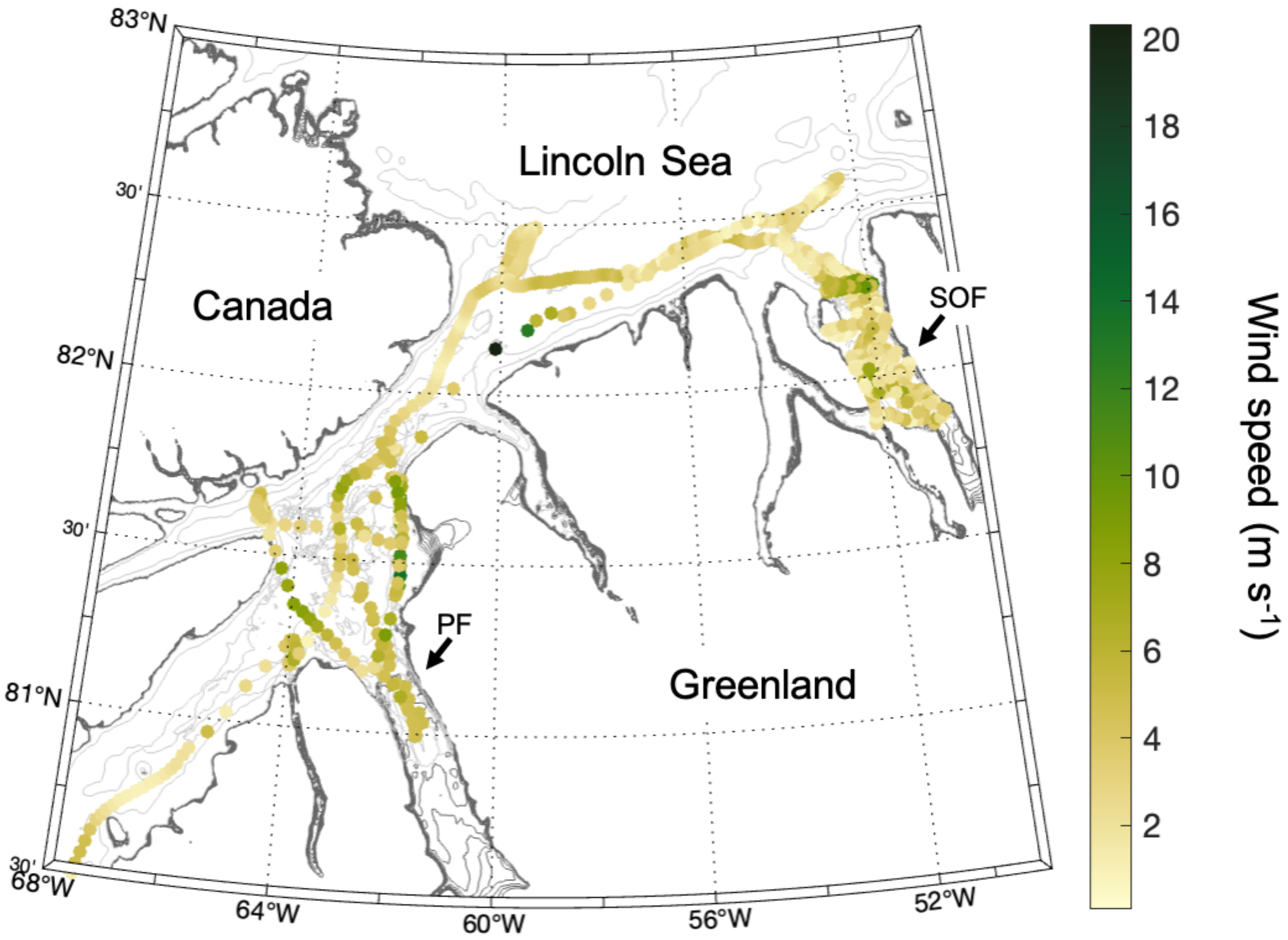

Figure 7. Spatial distribution of relative wind speed. PF corresponds to Petermann Fjord and SOF corresponds to Sherard Osborn Fjord.

Table 1. Observed and estimated values of the parameters used in the main manuscript and the uncertainties of the end-members used in the Monte-Carlo experiments of equations to compute the contributions of each driver controlling the pCO_2 changes (ΔpCO_2^T, ΔpCO_2^B, ΔpCO_2^{FW} and ΔpCO_2^R).

	Nares Strait	Lincoln Sea	Sherard Osborn Fjord	Petermann Fjord
Surface salinity (g kg^{-1})	29.85±0.41	27.29±3.67	18.1±1.19	29.88±0.42
Surface temperature (°C)	-0.49±0.35	-0.48±0.25	2.63±1.17	-0.17±0.27
Chlorophyll a (RFU)	1.49±1	0.86±0.55	0.29±0.1	0.53±0.3
Surface pCO_2 (μatm)	278±20	260±23	257±32	284±5
summer MLD depth (m)	12±6	11±3	6±0.2	11±3
TML depth (m)	41±9	55±11	43±4	33±8
TML salinity (g kg^{-1})	31.95±0.62	31.86±0.46	31.45±0.21	31.86±0.46
TML temperature (°C)	-1.46±0.12	-1.6±0.04	-1.53±0.05	-1.27±0.07
TML pCO_2 (μatm)	438±126	430±111	369±13	363±19
DIC freshwater (μmol kg^{-1})	992	1018	518	986
TA freshwater (μmol kg^{-1})	1045	1077	525	1040
Uncertainties				
Surface pCO_2 (μatm)	21	19	18	18
DIC freshwater (μmol kg^{-1})	83	56	32	33
TA freshwater (μmol kg^{-1})	33	36	32	24
